# Synthesis and Characterization of Bis[(*R* or *S*)-*N*-1-(X-C_6_H_4_)ethyl-2-oxo-1-naphthaldiminato-κ^2^*N*,*O*]-Λ/Δ-cobalt(II) (X = H, *p*-CH_3_O, *p*-Br) with Symmetry- and Distance-Dependent Vibrational Circular Dichroism Enhancement and Sign Inversion

**DOI:** 10.1021/acs.inorgchem.1c01503

**Published:** 2021-09-03

**Authors:** Marcin Górecki, Mohammed Enamullah, Mohammad Ariful Islam, Mohammad Khairul Islam, Simon-Patrick Höfert, Dennis Woschko, Christoph Janiak, Gennaro Pescitelli

**Affiliations:** † Department of Chemistry and Industrial Chemistry, University of Pisa, Pisa 56126, Italy; ‡ Institute of Organic Chemistry, Polish Academy of Sciences, Warsaw 01-224, Poland; ⊥ Department of Chemistry, Jahangirnagar University, Dhaka 1342, Bangladesh; ∥ Institute of Inorganic Chemistry and Structural Chemistry, Heinrich-Heine-University of Düsseldorf, Düsseldorf 40225, Germany

## Abstract

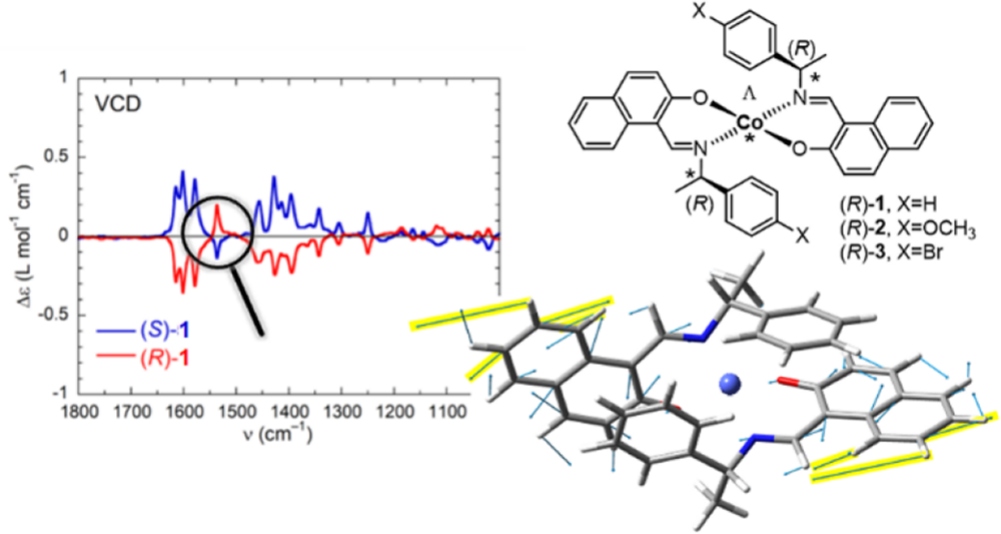

The enantiopure Schiff bases (*R* or *S*)-*N*-1-(X-C_6_H_4_)ethyl-2-hydroxy-1-naphthaldimine {X = H [(*R* or *S*)-HL1], *p*-CH_3_O [(*R* or *S*)-HL2], and *p*-Br [(*R*- or *S*)-HL3]} react with cobalt(II) acetate to give bis[(*R* or *S*)-*N*-1-(X-C_6_H_4_)ethyl-2-oxo-1-naphthaldiminato-κ^2^*N*,*O*]-Λ/Δ-cobalt(II) {X = H [Λ/Δ-Co-(*R* or *S*)-L1], *p*-CH_3_O [Λ/Δ-Co-(*R* or *S*)-L2], and *p*-Br [Λ/Δ-Co-(*R* or *S*)-L3]} (**1**–**3**), respectively. Induced Λ and Δ chirality originates at the metal center of the *C*_2_-symmetric molecule in pseudotetrahedral geometry. Differential scanning calorimetry analyses explored the thermal stability of the complexes, which undergo reversible phase transformation from crystalline solid to isotropic liquid phase for **1** and **3** but irreversible phase transformation for **2**. Like other cobalt(II) complexes, compounds **1**–**3** exhibit a continuous ensemble of absorption and circular dichroism bands, which span from the UV to IR region and can be collected into a superspectrum. Infrared vibrational circular dichroism (IR-VCD) spectra witness the coupling between Co^2+^-centered low-lying electronic states and ligand-centered vibrations. The coupling produces enhanced and almost monosignate VCD spectra, with both effects being mode-dependent in terms of the *A* or *B* symmetry (in the *C*_2_ point group) and distance from the Co^2+^ core.

## Abstract 2

Chiroptical superspectra of naphthaldiminatocobalt(II) complexes **1**−**3** exhibit a continuous ensemble of absorption and circular dichroism bands from the UV to IR region, including peculiar and almost monosignate vibrational circular dichroism (VCD) spectra dominated by the coupling between Co^2+^-centered low-lying electronic states and ligand-centered vibrations, which depend on the normal-mode symmetry and distance from the Co^2+^ core

## Introduction

**Scheme 1:**
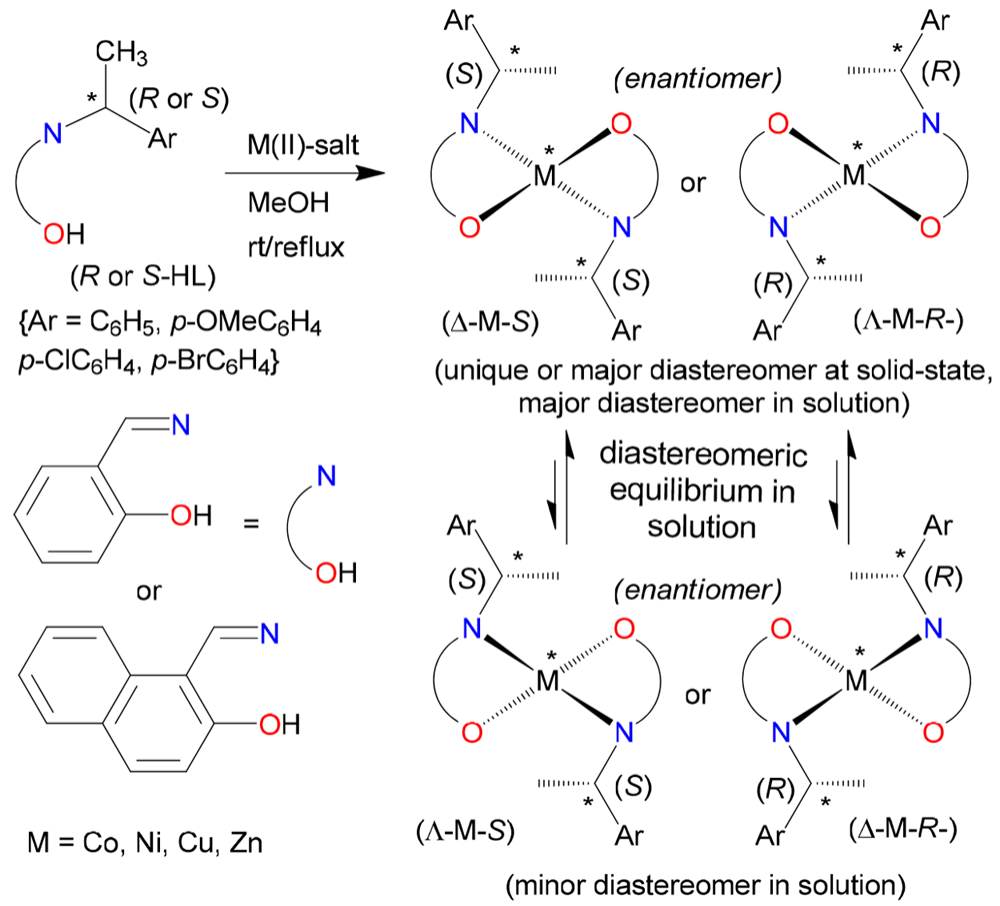
Diastereoselection and Enantiomeric Configuration At-Metal of Nonplanar Bis(N,O-chelate) Schiff Base Complexes Viewed along the *C*_2_ Axis (Perpendicular to the Paper Plane): Λ as the Left-Handed Helicity, Δ as the Right-Handed Helicity along the *C*_2_ Axis, for Complexes of Cobalt(II),^19^ Nickel(II),^21,23^ Copper(II),^20,22,25^ and Zinc(II)^24,26^ For some copper complexes, the sign of induction is reversed (see the text).^22^

Transition-metal(II) complexes with achiral/chiral Schiff bases are of continued interest in the context of their molecular structures, variable coordination geometry, chiroptical properties, Λ versus Δ chirality induction-at-metal, and concomitant (dia)stereoselectivity.^1−18^ Two bidentate Schiff base ligands can coordinate to divalent metal(II) ions (Mn, Fe, Co, Ni, Cu, and Zn) and provide nonplanar *C*_2_-symmetrical complexes with the formula M(N,O)_2_ (N,O = deprotonated Schiff base) with distorted tetrahedral/square-planar geometry. Such a coordination of two Schiff base ligands leads to chirality induction-at-metal and gives right Δ- and left Λ-handed metal configurations (Scheme 1). If enantiopure (*R* or *S*) Schiff base ligands are used, then two diastereomers Λ-M-*R* and Δ-M-*R* (or Δ-M-*S* and Λ-M-*S*) will form.^19−25^ The various noncovalent *inter*- and/or *intra*molecular interactions at the solid state and solute–solvent interactions in solution result in a free energy difference between the two diastereomers; thereby one of the diastereomers is thermodynamically favored (i.e., unique or major diastereomer). The ligand chirality and design, substituents, steric constraints, metal ion and counteranion selection, reaction conditions, crystallization protocol, etc., can significantly control this phenomenon.

Our recent studies on divalent transition-metal complexes with enantiopure Schiff base ligands (*R* or *S*)-*N*-1-(Ar)ethylsalicylaldimine/-2-hydroxy-1-naphthaldimine (metal = Co,^19^ Ni,^21,23^ Cu,^20,22,25^ Zn,^24,26^ Rh(η^4^-cod);^27,28^ Ar = C_6_H_5_, *p*-CH_3_OC_6_H_4_, *p*-ClC_6_H_4_, *p*-BrC_6_H_4_) in the context of diastereoselection and chirality induction-at-metal demonstrated the formation of a unique or major diastereomer Λ-M-*R* or Δ-M-*S* with distorted tetrahedral/square-planar geometry in the solid state (Scheme 1). This was confirmed by X-ray structure determination, the most reliable method to assign the absolute configuration of the metal center, of a single investigated enantiopure crystal at the solid state. The limitation of this assignment is that for X-ray measurements, usually, one single crystal is chosen, and the conclusion drawn about the existence of a unique isomer does not exclude the presence of other isomers (may be minor) in the bulk sample. In this connection, differential scanning calorimetry (DSC) analyses of Zn-*N*-1-(aryl)ethyl-2-oxo-1-naphthaldiminate in the solid state showed the presence of both Λ and Δ diastereomers at a ratio of ca. 81:19 (Δ:Λ) in the bulk sample for the *S* enantiomer and ca. 85:15 (Λ:Δ) for the *R* enantiomer.^26^ Indeed, both diastereomers (Δ and Λ) also coexist in a single enantiopure crystal (eutectic mixture) of Cu^II^-(*R*)-*N*-1-(*p*-CH_3_OC_6_H_4_)ethyl-2-oxo-1-naphthaldiminate.^20^ However, the preferred formation of one diastereomer is different in solution (or the gas phase) and the solid state because in solution diastereomeric equilibria with Λ/Δ helicity inversion at-metal may happen. In fact, solid versus solution studies revealed solvation-induced helicity inversion from Λ-M-*R* or Δ-M-*S* (solid state) to Δ-M-*R* or Λ-M-*S* (solution) for bis[*N*-1-(*p*-CH_3_OC_6_H_4_)ethylsalicylaldiminato-κ^2^*N*,*O*]copper(II) derivatives^22^ and bis[(*R* or *S*)-*N*-1-phenylethyl-2,4-dihalosalicylaldiminato-κ^2^*N*,*O*]copper(II),^25^ as evidenced by combined studies on the experimental and simulated electronic circular dichroism (ECD) spectra in solution. Solution studies further explored the existence of a dynamic diastereomeric equilibrium between the two diastereomers (Λ ⇆ Δ), controlled by the temperature, as examined by variable-temperature ^1^H NMR and ECD spectra.^19,26^

**Scheme 2:**
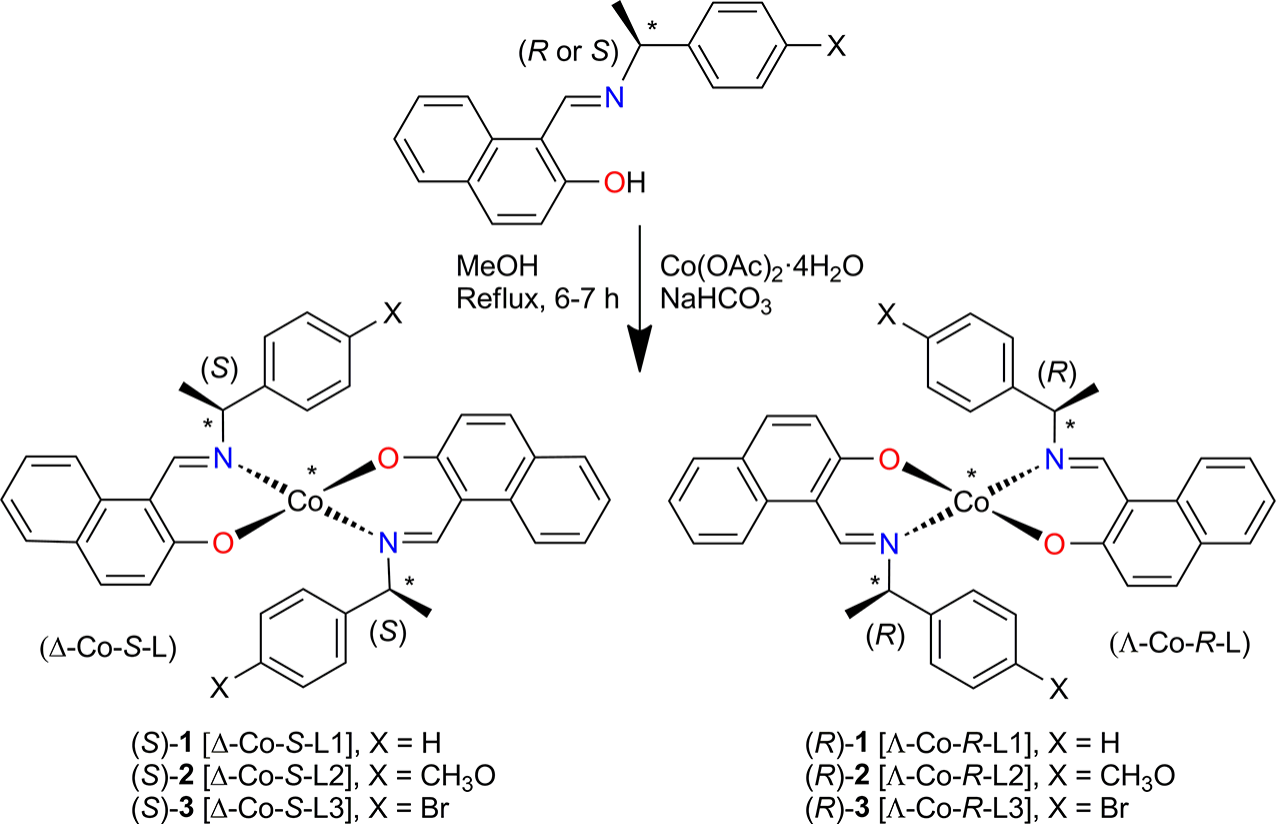
Synthetic Route of Bis[(*R* or *S*)-*N*-1-(X-C_6_H_4_)ethyl-2-oxo-1-naphthaldiminato-κ^2^*N*,*O*]-Λ/Δ-cobalt(II) [Λ/Δ-Co-(*R* or *S*)-L, **1**–**3**] Showing Induced Chirality At-Metal Center with Δ (Left)- and Λ (Right)-Handed Diastereomers in *C*_2_-Symmetrical Pseudotetrahedral Geometry For a given ligand *R* or *S* configuration, the found chirality at-metal is shown by Λ or Δ based on the solid-state single-crystal X-ray structure.

In the present paper, we report the synthesis, X-ray structure determination, DSC analysis, and thorough spectroscopic characterizations of bis[(*R* or *S*)-*N*-1-(X-C_6_H_4_)ethyl-2-oxo-1-naphthaldiminato-κ^2^*N*,*O*]-Λ/Δ-cobalt(II) (**1**–**3**, Scheme 2). Apart from the motivation dictated by a further extension of the family of cobalt(II) Schiff base complexes, a second main interest in compounds **1**–**3** is related to their spectroscopic properties. Thanks to the presence of multiple aromatic chromophores and of the Co^II^ center, compounds **1**–**3** are amenable to chiroptical characterization over a very broad range of the electromagnetic spectrum covering the UV, visible, near-infrared (NIR), and IR regions, where respectively UV–vis ECD, NIR-CD, and vibrational circular dichroism (VCD) may be observed. In our previous work on the salicylaldiminato Schiff base analogues of **1**–**3**, we introduced the concept of a *(chiro)optical superspectrum* to describe a continuous set of optical and chiroptical (CD) spectra spanning the aforementioned regions.^19^ The superspectrum is rich in several bands and offers a distinctive fingerprint of the structure and stereochemistry of the metal complexes. The (chiro)optical superspectrum only manifests in the presence of the Co^II^ core, which justifies the extension to cobalt(II) compounds **1**–**3** of previous studies concerning the homoleptic series of copper(II), nickel(II), and zinc(II).^20,21,26^ Particularly significant are the NIR and IR regions, where Co^II^-centered transitions occur, allied with d^7^ electronic configuration in a distorted square-planar geometry. In the NIR and IR ranges, these transitions are endowed with relatively large dissymmetry *g* factors (Δε/ε) and report the chirality at-metal. In the IR region around 3000 cm^–1^, moreover, they uniquely overlap with ligand-centered vibrational transitions, allowing one to observe the effects of strong vibronic coupling between ground-state vibrational transitions and magnetic-dipole allowed low-lying electronic excited states (LLESs).^29,30^ These effects extend to the middle IR (fingerprint) region, where two facts happen. First, the VCD spectrum, which is normally composed of bands with alternating positive and negative signs, becomes almost monosignate (with a certain sign for a given enantiomer).^19^ Second, the intensity of the VCD bands is enhanced by a factor up to ∼10, a circumstance that helps VCD characterization because VCD spectra are often allied with intrinsically weak signals.^31,32^ While LLES-related VCD enhancement had been observed before for many different transition-metal complexes,^30,33−44^ the monosignate appearance of VCD spectra was unprecedented. Some of us have recently demonstrated that the two phenomena are interrelated, but the latter one is also symmetry-dependent; that is, it needs *C*_2_-symmetric (and possibly higher-symmetry) compounds to be observed.^45^ Therefore, the current series of compounds **1**–**3** offered a good chance to verify whether the previous results, observed for the salicylaldiminato Schiff base analogues, could be reproduced with a similar, although different and larger, ligand. We indeed obtained, in the present case too, a set of (chiro)optical superspectra that uniquely characterize the investigated metal compounds **1**–**3**, including the peculiar bands in the NIR region and in the IR region around 3000 cm^–1^. In addition, we detected a significant difference in the VCD spectrum, where the monosignate appearance is interrupted by a moderately intense band at 1536–1538 cm^–1^, for which a theoretical interpretation is discussed.

## Results and Discussion

### Synthesis and Characterization

The enantiopure Schiff bases (*R* or *S*)-*N*-1-(X-C_6_H_4_)ethyl-2-hydroxy-1-naphthaldimine {X = H [(*R* or *S*)-HL1], *p*-CH_3_O [(*R* or *S*)-HL2], and *p*-Br [(*R* or *S*)-HL3]} react with cobalt(II) acetate in the presence of NaHCO_3_ under reflux and provide bis[(*R* or *S*)-*N*-1-(X-C_6_H_4_)ethyl-2-oxo-1-naphthaldiminato-κ^2^*N*,*O*]-Λ/Δ-cobalt(II) (**1**–**3**) {X = H [Λ/Δ-Co-(*R* or *S*)-L1], *p*-CH_3_O [Λ/Δ-Co-(*R* or *S*)-L2], and *p*-Br [Λ/Δ-Co-(*R* or *S*)-L3]}, respectively (Scheme 2). The IR spectra of the complexes show the main characteristic bands at ca. 1616 and 1601 cm^–1^ for the ν(C=N) stretching vibration. Electron impact ionization mass spectra (EI-MS) show the parent ion peak ([M]^+^) at *m*/*z* 607 for Λ/Δ-Co-(*R* or *S*)-L1 (**1**), *m*/*z* 667 for Λ/Δ-Co-(*R* or *S*)-L2 (**2**), and *m*/*z* 765 for Λ/Δ-Co-(*R* or *S*)-L3 (**3**). The spectra show the ion peaks for the monoligated species at *m*/*z* 332 ([CoL1-H]^+^), 362 ([CoL2-H]^+^), and 410 ([CoL3-H]^+^). The spectra are further dominated by the ion peaks for the ligands at *m*/*z* 275 ([HL1]^+^), 305 ([HL2]^+^), and 353 ([HL3]^+^) (Figure S1).

### X-ray Structural Analyses

**Figure 1:**
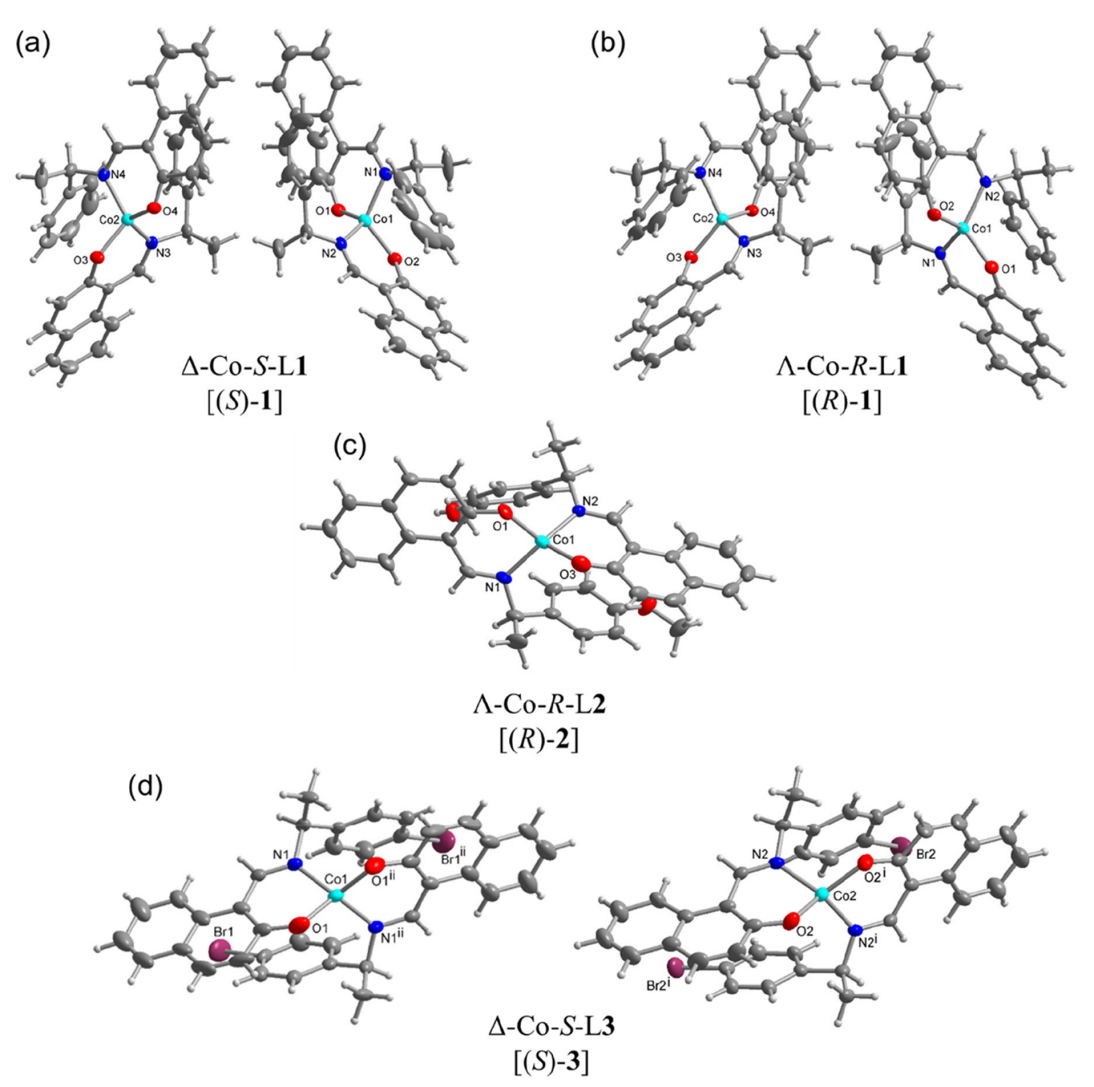
X-ray molecular structures of (a) (*S*)-**1**, (b) (*R*)-**1**, (c) (*R*)-**2**, and (d) (*S*)-**3**, with the symmetry-independent molecules shown. Thermal ellipsoids at 50% (H atoms at arbitrary radii). See Figures S2–S5 for the individual images of the symmetry-independent molecules with full atom-numbering schemes. Symmetry labels for (*S*)-**3**: i = *y*, *x*, 1 – *z*; ii = 2 – *x*, 1 – *x* + *y*, ^2^/_3_ – *z*.

Single-crystal X-ray molecular structures for **1**–**3** are shown in Figure 1. Crystal data and structure refinements are reported in Table S1. Selected bond lengths and bond angles are listed in Tables S2 and S3, which are comparable to the homoleptic bis[(*R* or *S*)-*N*-1-(X-C_6_H_4_)ethyl-2-oxo-1-naphthaldiminato-κ^2^*N*,*O*]-Λ/Δ-M(II) (M = Cu,^20^ Ni,^21^ Zn;^26^ X = H, *p*-CH_3_O, *p*-Br) complexes. The complexes crystallize in noncentrosymmetric space groups of *P*1 for Δ-Co-*S*-L1 [(*S*)-**1**] and Λ-Co-*R*-L1 [(*R*)-**1**], *P*2_1_ for Λ-Co-*R*-L2 [(*R*)-**2**], and *P*3_2_21 for Δ-Co-*S*-L3 [(*S*)-**3**]. The molecular structure determinations reveal that the Co ion is four-coordinated by two phenolate O and two imine N atoms from two Schiff base ligands, leading to a N_2_O_2_ coordination sphere in pseudotetrahedral geometry. The two coordinated ligands are crystallographically independent in (*S*)-**1**, (*R*)-**1**, and (*R*)-**2**, and the Co atom sits in a general position. Both ligands present an approximately *C*_2_-symmetric arrangement (cf. Scheme 2) with the assumed *C*_2_-axis bisecting the O–Co–O and N–Co–N angles. Looking along the *C*_2_ axis in (*S*)-**1**, (*R*)-**1**, (*R*)-**2**, and (*S*)-**3** passing through the Co center perpendicular to the O–(Co)–O and the N–(Co)–N edges, the absolute configuration Δ or Λ form is determined by the orientation of the chelate ring planes. For the Λ form, the chelate ring planes are oriented as propeller blades that form a left-handed helix, and for the Δ form, it is a right-handed helix (Scheme 1). The metal-centered chirality or absolute configuration Δ or Λ form is denoted as part of the complex’s designation. For a given ligand (*S*)- or (*R*)-HL chirality, only one Δ- or Λ-configuration at-metal center is found in each investigated crystal based on the absolute structure or Flack parameter values of 0.007–0.050 (Table S1).^46−49^ In fact, Flack parameter values close to zero and other refinement parameters (e.g., normal atom temperature factors and the absence of any molecular disorder) rule out any significant amount of molecule with an opposite chirality at-metal center.^46−49^ This means that there is no diastereomeric mixture (i.e., coexistence of both Δ-Co and Λ-Co) within one of the investigated enantiopure single crystals. In contrast, a diastereomeric mixture with both the Λ and Δ forms in a single enantiopure crystal of Cu^II^-(*R*)-*N*-1-(*p*-CH_3_OC_6_H_4_)ethyl-2-oxo-1-naphthaldiminate was reported.^20^

It is worth noting that there are two symmetry-independent molecules (A and B) in (*S*)-**1**, (*R*)-**1**, and (*S*)-**3**. Also, the homoleptic nickel, copper, and zinc structures with L1 or L2 ligands contain two symmetry-independent molecules in the asymmetric unit.^20,21,26^ In (*S*)-**3**, the asymmetric unit contains two halves of two symmetry-independent molecules that lie on a crystallographic 2-fold axis passing through the Co centers (Figure 1). The Co–O bond lengths are always shorter by ∼0.05 Å than the Co–N bonds. This reflects the stronger attraction between the negatively charged phenolate oxygen anion (O^–^) and the positively charged Co^II^ cation (Tables S2 and S3). The imine bond (C=N) lengths are around 1.3 Å, in agreement with the double-bond character.

The observed Δ-Co-*S* or Λ-Co-*R* configuration in the solid state is supposed to be induced diastereospecifically by the conformational preference of the coordinated N,O-chelate rings, resulting from minimum steric and/or chelation requirements (leading to thermodynamic stability) by the ligand chirality and/or substituents on the aryl ring. These results are in parallel to the preferred solid-state formation of Λ-M-*R* or Δ-M-*S* (unique or major diastereomers) in the homoleptic bis[(*R* or *S*)-*N*-1-(X-C_6_H_4_)ethyl-2-oxo-1-naphthaldiminato-κ^2^*N*,*O*]-Λ- or Δ-M(II) (Table 1)^20,21,26^ and the analogous bis[(*R* or *S*)-*N*-1-(*p*-X-C_6_H_4_)ethylsalicylaldiminato-κ^2^*N*,*O*]-Λ- or Δ-M(II) (M = Co, Cu, Ni, Zn; X = H, *p*-CH_3_O, *p*-Cl/Br; Table S4),^19,22−24^ as evidenced by X-ray structural analyses. On the contrary, copper(II) complexes with (*R* or *S*)-*N*-1-(*p*-X-C_6_H_4_)ethylsalicylaldimine (X = H, *p*-Cl/Br) exhibit the oppositely configured Δ-M-*R* or Λ-M-*S* diastereomers in the solid state.^22^ With this exception, the phenomena of diastereoselection and chirality induction-at-metal in the solid state are, in general, solely controlled by the ligand *S* or *R* chirality and are independent of the ligand substituents or modification and/or metal-ion selection. Solution studies also support this notion with diastereomeric excess of Δ-Co-*S* or Λ-Co-*R* in the *S*- or *R*-ligated complexes (Tables 1 and S4).

**Table 1:** Ligand Chirality and Substituents Leading to Chirality Induction-At-Metal in Bis[(*R* or *S*)-*N*-1-(*p*-X-C_6_H_4_)ethyl-2-oxo-1-naphthaldiminato-κ^2^*N*,*O*]-Λ- or Δ-M(II) in the Solid State and Solution

complex [M-(*R* or *S*-L)]	ligand substituent (X)	ligand chirality → induction-at-metal in the solid state	ligand chirality → induction-at-metal in solution	ref
M = Co	H (A/B)^a^	*S* → Δ, *R* → Λ	*S* → Δ, *R* → Λ	this work
	*p*-CH_3_O	*R* → Λ	*R* → Λ	
	*p*-Br (A/B)^a^	*S* → Δ	*S* → Δ	
M = Cu	H	*S* → Δ, *R* → Λ	*S* → Δ, *R* → Λ	(20)
	*m*-CH_3_O^b^	*R* → Λ/Δ		
	*p*-Br	*R* → Λ	*R* → Λ	
M = Ni	H (A/B)^a^	*S* → Δ, *R* → Λ	*S* → Δ, *R* → Λ	(21)
M = Zn	H (A/B)^a^	*S* → Δ, *R* → Λ	*S* → Δ, *R* → Λ	(26)
	*p*-CH_3_O (A/B)^a^	*S* → Δ, *R* → Λ	*S* → Δ, *R* → Λ	

a Two symmetry-independent molecules A and B in an asymmetric unit with a single diastereomer (Λ or Δ).

b Diastereomeric mixture with opposite Λ and Δ configurations in a single enantiopure crystal.

For quantitative assessment of the coordination geometry around the metal ion, the degree of distortion from tetrahedral to square-planar can be determined by the dihedral angle θ (deg) between the two coordinating planes N1–Co–O1 and N2–Co–O2, by its normalized function τ_tet-sq_ (=θ/90°), or by the geometry index τ_4_.^19−24^ The values of the degree of distortion in the present complexes and in the homoleptic copper,^20^ nickel,^21^ and zinc^26^ complexes are listed in Table 2. These values are close to ideal tetrahedral for cobalt and zinc complexes and to ideal square-planar for nickel and copper complexes. Indeed, the degree of distortion is slightly influenced by the substituents on the aryl ring (X = H, CH_3_O, and Br) in each group of complexes (Table 2) due to steric constraints experienced in the coordination sphere. Although the degree of distortion is substantially changed from tetrahedral (for Co and Zn) to near square-planar (for Ni and Cu) geometry, no influences on diastereoselection and chirality induction-at-metal (i.e., Λ-M-*R* or Δ-M-*S*) are so far observed.

**Table 2:** Measurements of the Distortion from Tetrahedral to Square-Planar Geometry in Bis[(*R* or *S*)-*N*-1-(*p*-X-C_6_H_4_)ethyl-2-oxo-1-naphthaldiminato-κ^2^*N*,*O*]-Λ- or Δ-M(II)

complex [M-(*R* or *S*)-L]	ligand substituent (X)	ligand chirality	Δ or Λ chirality induction	θ/deg^b^	τ_tet-sq_ = θ/90°	τ_4_^c^	ref
M = Co	H (A/B)^a^	(*S*)-L1	Δ	88.14/88.52	0.98/0.98	0.83/0.82	this work
		(*R*)-L1	Λ	87.95/88.57	0.98/0.98	0.83/0.82	
	*p*-CH_3_O	(*R*)-L2	Λ	82.36	0.92	0.80	
	*p*-Br (A/B)^a^	(*S*)-L3	Δ	84.76/82.63	0.94/0.92	0.83/0.81	
M = Cu	H	(*S*)-L1	Δ	27.0	0.30	0.27	(20)
		(*R*)-L1	Λ	26.5	0.29	0.27	
	*p*-Br	(*R*)-L3	Λ	3.88	0.04	0.04	
M = Ni	H (A/B)^a^	(*S*)-L1	Δ	9.98/1.73	0.11/0.02	0.10/0.02	(21)
		(*R*)-L1	Λ	10.00/1.80	0.11/0.02	0.10/0.02	
M = Zn	H (A/B)^a^	(*S*)-L1	Δ	88.78/88.03	0.99/0.98	0.84/0.83	(26)
		(*R*)-L1	Λ	88.89/88.24	0.99/0.98	0.83/0.83	
	*p*-CH_3_O (A/B)^a^	(*S*)-L2	Δ	83.37/88.45	0.93/0.98	0.83/0.83	
		(*R*)-L2	Λ	88.54/83.48	0.98/0.93	0.83/0.83	

a Two symmetry-independent molecules with A and B in an asymmetric unit with a single diastereomer (Λ or Δ).

b Dihedral angle between the two coordinating planes N1–Co–O1 and N2–Co–O2.

c τ_4_ = [360° – (α + β)]/141°, where α and β are the two largest angles (N–Co–N and O–Co–O) in the four-coordinate species;^50^ see Tables S2 and S3.

### Thermal Analyses and Phase Transformation

**Figure 2:**
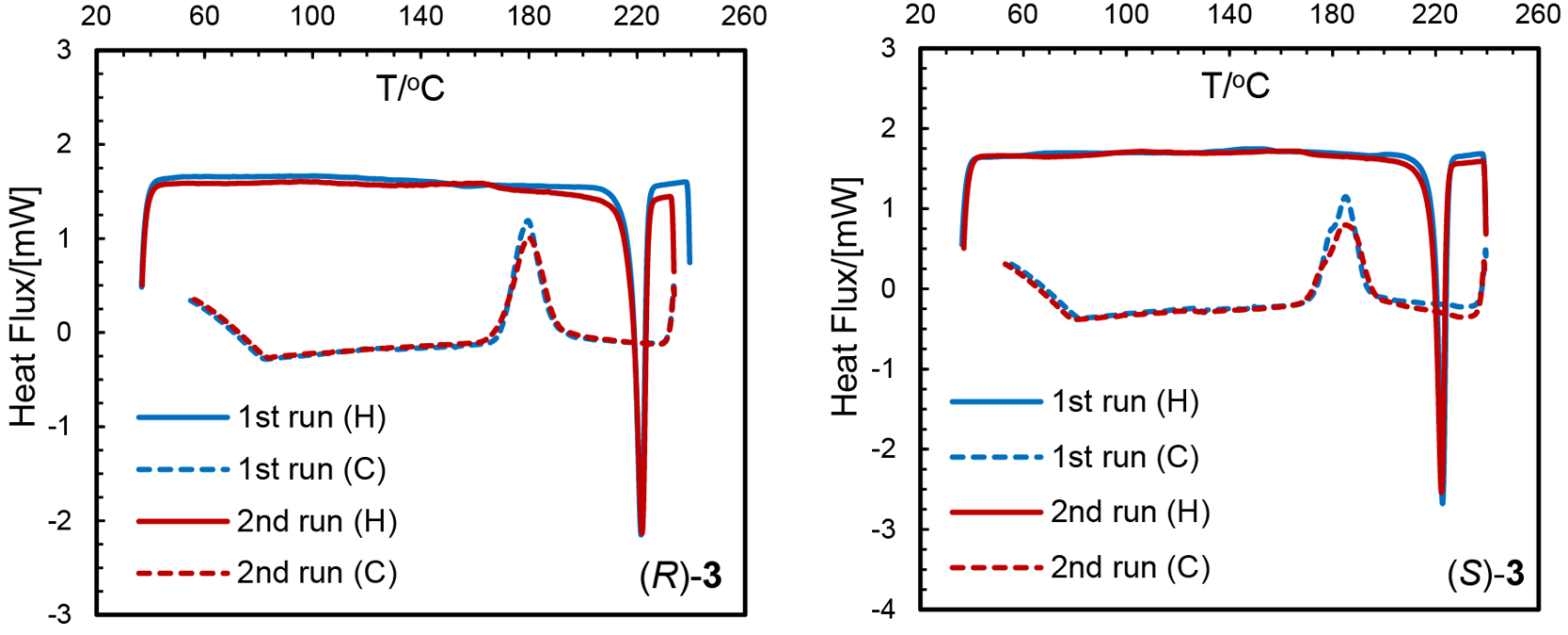
DSC curves for complexes (*R*)- and (*S*)-**3** (H/C = heating/cooling).

Thermally induced structural phase transformations have been reported for transition-metal chiral N,O-chelate Schiff base complexes, accompanying a change from the solid state at low temperature to the isotropic liquid phase at high temperature.^11,17,19−21,23,51−53^ DSC analyses explore the thermal stability of the complexes and have successfully been used to study such thermally induced structural phase transformation behavior in detail. DSC heating curves for the present complexes show an endothermic peak with a considerable amount of heat of transformation (Δ*H*/kJ mol^–1^) at 178–181 °C for (*R*)/(*S*)-**1**, 132–135 °C for (*R*)/(*S*)-**2**, and 221–223 °C for (*R*)/(*S*)-**3** (Table 3 and Figures 2 and S6). The cooling curves show exothermic peaks in the reverse direction at relatively low temperature, except for (*R*)/(*S*)-**2**. DSC analyses repeated for the same sample (probe) in the second run (cycle) produced identical results (Figures 2 and S6 and Table 3), which suggest the absence of any decomposition of the complexes and hence their thermal stability under the present experimental conditions. Thermal stability (transformation temperature) increases with increasing mass of the complexes from X = H (178–181 °C) to X = *p*-Br (221–223 °C), while it decreases for X = *p*-CH_3_O (132–135 °C) possibly because of the extra flexibility (methoxy group rotamerism). DSC results thus demonstrate a thermally induced reversible phase transformation from the crystalline solid to the isotropic liquid phase for (*R*)/(*S*)-**1** and (*R*)/(*S*)-**3**. Similar results were found for the analogous cobalt(II) salicylaldimine^19^ and zinc(II) naphthaldimine^26^ complexes. On the contrary, the cooling curves for (*R*)/(*S*)-**2** show no peaks in the reverse direction, suggesting an irreversible phase transformation. The heating curves for **1** and **2** show a weak broad peak below 70 °C in the first run due to the presence of a small amount of solvent in the sample, which evaporates upon further heating, and the peak disappears in the second run. DSC curves for the free Schiff base ligand (*R*)-HL2 are shown in Figure S6, which displays an endothermic peak at 105 °C, corresponding to an irreversible phase transformation. A comparison of the DSC curves for the free Schiff base ligand and complexes suggests no free ligand in the samples. The presence of a single peak (both for the heating and cooling curves) indicates a single diastereomer of Λ-Co-(*R*)-L or Δ-Co-(*S*)-L in the solid state, in accordance with the X-ray analyses for all compounds **1**–**3**.

**Table 3:** Thermal Analyses (DSC) Data for Λ-Co-(*R*)-L or Δ-Co-(*S*)-L Complexes^a^

complex	heating curve peak temp (°C)/Δ*H*^b^	cooling curve peak temp (°C)/Δ*H*^b^
(*R*)-**1**	179.6/–31.0 (1st run)	132.1/13.6 (1st run)
	178.3/–28.9 (2nd run)	137.2/20.0 (2nd run)
(*S*)-**1**	178.9/–25.9 (1st run)	139.5/19.1 (1st run)
	180.4/–24.2 (2nd run)	161.6/21.0 (1st run)
(*R*)-**2**	135.1/–19.7	no peak
(*S*)-**2**	132.6/–17.7	no peak
(*R*)-HL2	105.3/–21.7	no peak
(*R*)-**3**	223.4/–33.5 (1st run)	179.6/28.8 (1st run)
	221.8/–31.9 (2nd run)	180.1/27.9 (2nd run)
(*S*)-**3**	222.7/–33.3 (1st run)	185.1/40.4 (1st run)
	222.3/–36.9 (2nd run)	185.1/48.1 (2nd run)

a DSC was run just before the decomposition temperature of the complexes.

b Δ*H* = heat of transformation (kJ mol^–1^).

### Chiroptical Characterization

**Figure 3:**
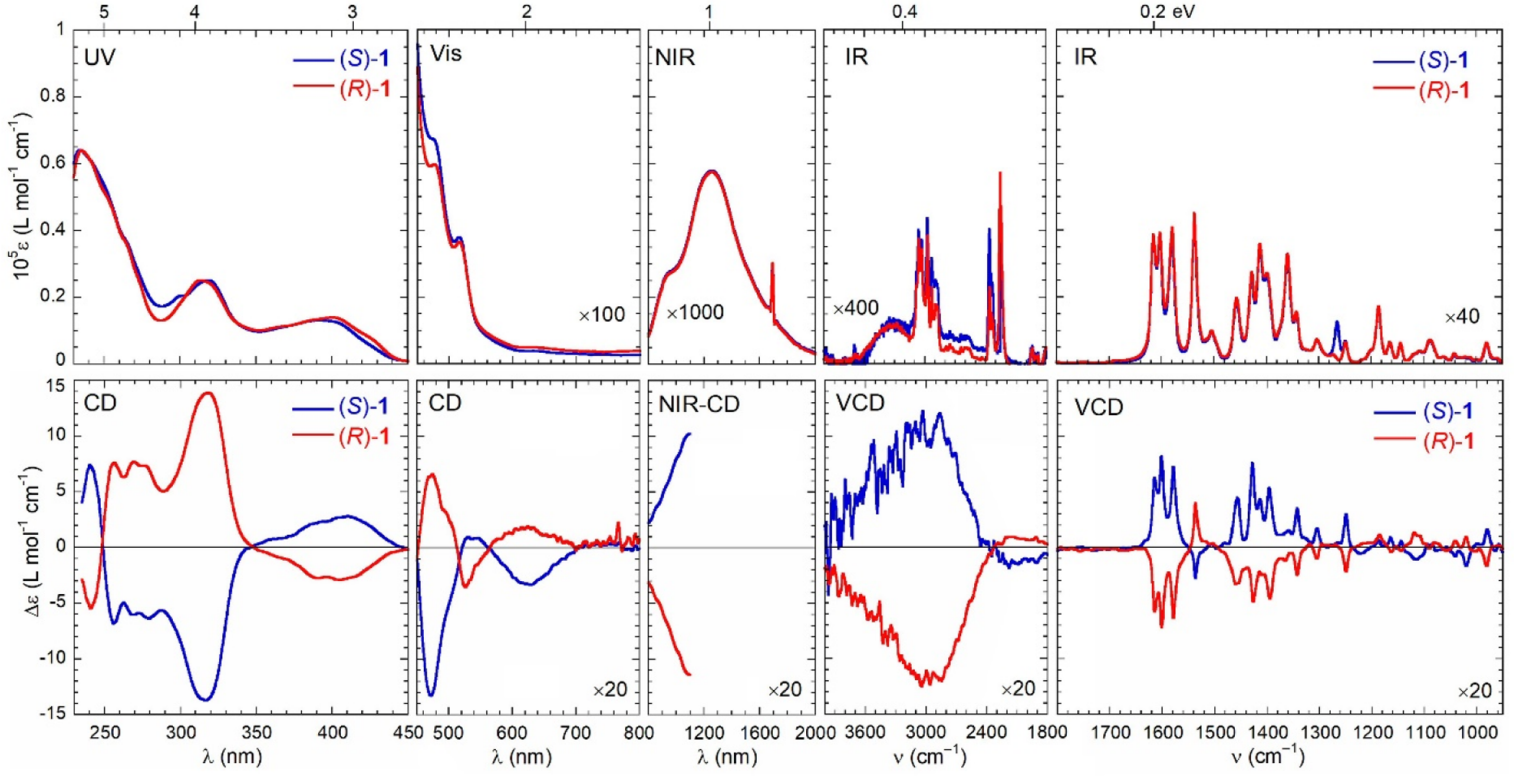
Optical (top) and chiroptical (bottom) superspectra of (*R*)-**1** (red curves) and (*S*)-**1** (blue curves). Measurement conditions: UV region, 0.27 mM in CHCl_3_, 0.1 cm cell; visible region, 0.27 mM in CHCl_3_, 1 cm cell; NIR region, 0.88 mM in CHCl_3_, 2 cm cell; VCD region, 4000–2000 cm^–1^ subrange, 0.11 M [(*R*)-**1**] and 0.12 M [(*S*)-**1**] in CDCl_3_, 200 μm cell; VCD region, 2000–900 cm^–1^ subrange, 40 mM in CDCl_3_, 200 μm cell. See Materials and Methods for further details.

**Figure 4:**
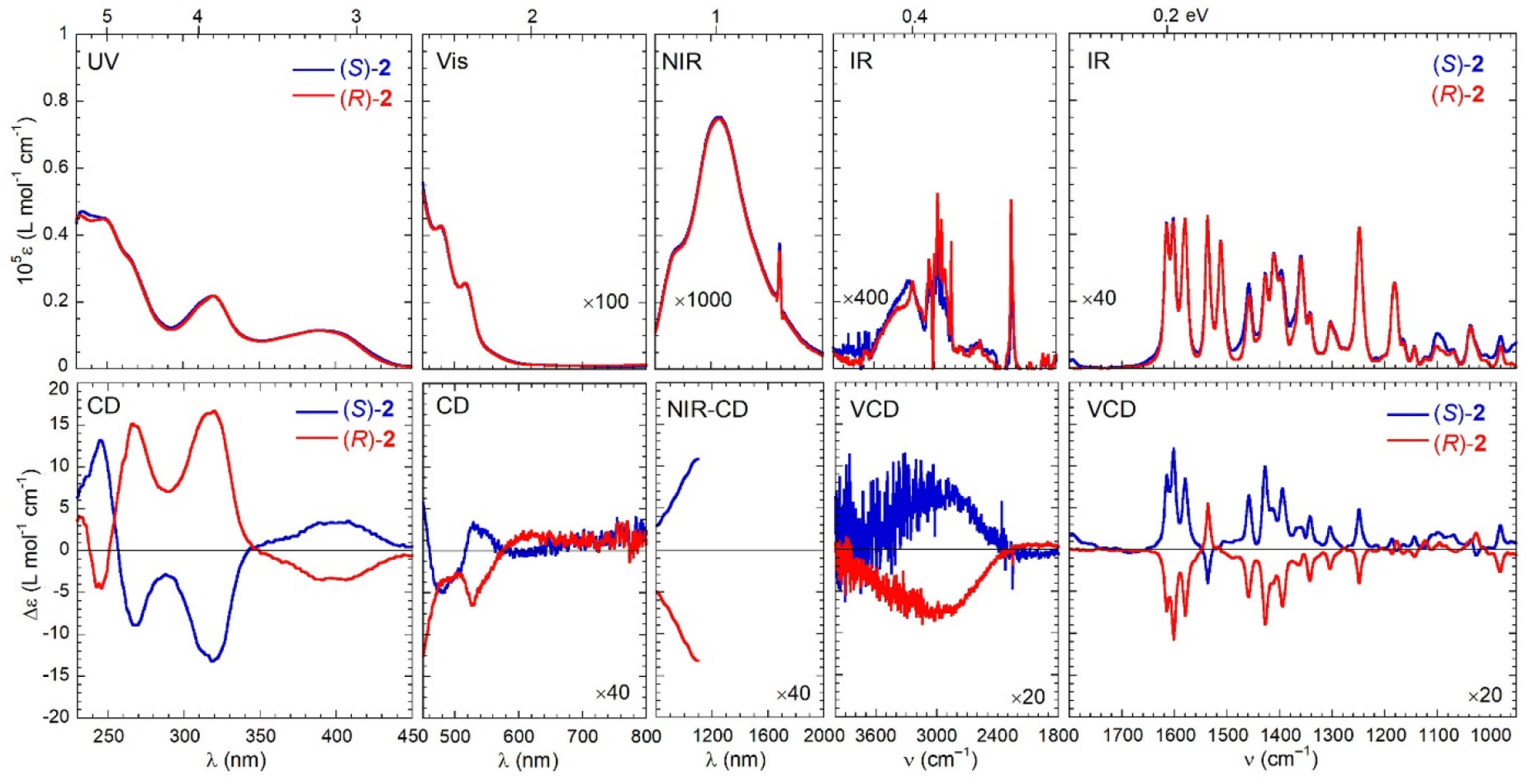
Optical (top) and chiroptical (bottom) superspectra of (*R*)-**2** (red curves) and (*S*)-**2** (blue curves). Measurement conditions: UV region, 0.25 mM [(*R*)-**2**] and 0.21 mM [(*S*)-**2**] in CHCl_3_, 0.1 cm cell; visible region, 0.25 mM [(*R*)-**2**] and 0.21 mM [(*S*)-**2**] in CHCl_3_, 1 cm cell; NIR region, 0.83 mM [(*R*)-**2**] and 0.89 mM [(*S*)-**2**] in CHCl_3_, 2 cm cell; VCD region, 4000–2000 cm^–1^ subrange, 0.14 M [(*R*)-**2**] and 0.05 M [(*S*)-**2**] in CDCl_3_, 200 μm cell; VCD region, 2000–900 cm^–1^ subrange, 32 mM in CDCl_3_, 200 μm cell. See Materials and Methods for further details.

**Figure 5:**
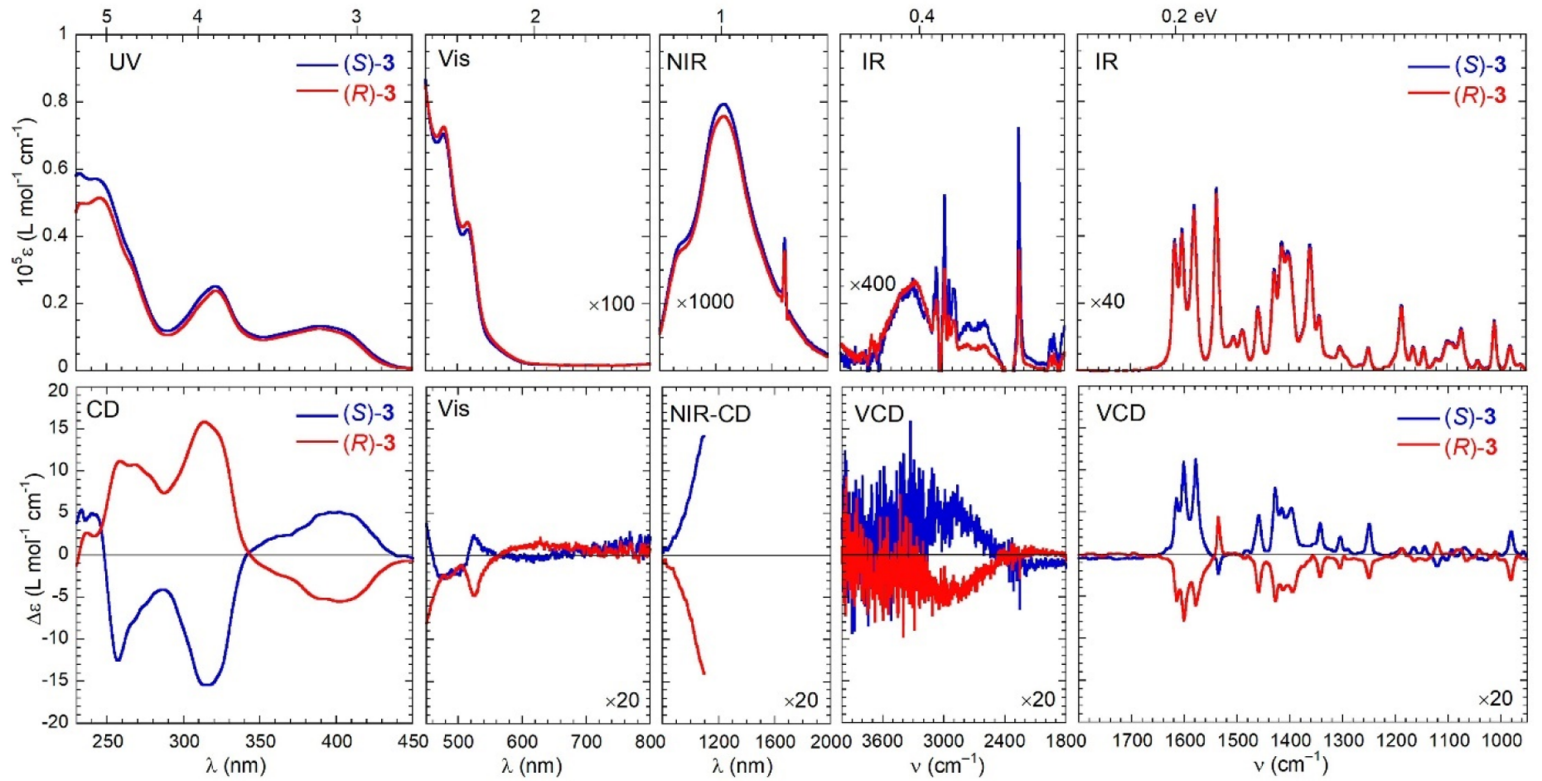
Optical (top) and chiroptical (bottom) superspectra of (*R*)-**3** (red curves) and (*S*)-**3** (blue curves). Measurement conditions: UV region, 0.17 mM in CHCl_3_, 0.1 cm cell; visible region, 0.17 mM in CHCl_3_, 1 cm cell; NIR region, 0.76 mM in CHCl_3_, 2 cm cell; VCD region, 4000–2000 cm^–1^ subrange, 0.06 M [(*R*)-**2**] and 0.04 mM [(*S*)-**2**] in CDCl_3_, 200 μm cell; VCD region, 2000–900 cm^–1^ subrange, 35 mM in CDCl_3_, 200 μm cell. See Materials and Methods for further details.

The (chiro)optical superspectra of compounds **1**–**3**, whose meaning was discussed in the Introduction, are shown in Figures 3–5. They are actually composed of five distinct spectra for the absorption and five for the circular dichroism, recorded on four different samples with varying concentration and cell path length on four different instruments. The experimental details are reported in Materials and Methods. Chloroform and chloroform-*d* were consistently used as the solvents. In each spectrum, we use the typical wavelength (in nm) and wavenumber (in cm^–1^) units for the UV–vis–NIR and IR regions, respectively; however, to emphasize the continuous pattern of transitions that occur for compounds **1**–**3**, the various spectra are plotted side by side without interruptions (although there is a gap in the experimentally accessible frequencies between the NIR and IR regions), and electronvolt values are also displayed on the top *x* axis of the spectra as a common energy reference. We can make the following general observations by visual inspection of the spectra: (a) for the two enantiomers of each compound, the absorption spectra are identical, while the CD spectra are specular, as expected; (b) the overall spectral profile is rather similar for the three compounds **1**–**3**, although some differences emerge in the UV and mid-IR (fingerprint) regions; (c) for the corresponding bands, the signs of the chiroptical spectra are consistent for a given absolute configuration of the ligand.

The same points applied, as well, for the previous series of salicylaldiminato Schiff base analogues.^19^ In the following, we describe in more detail the various spectral regions, emphasizing also the differences between the two series.

In the *UV region* (230–450 nm), the spectra are dominated by the transitions of the aromatic chromophore, mainly of the π–π* type. Three main UV bands are observed with maxima at 230–240 (this band is split for **2** and **3**), 315–320, and 390–395 nm. They are all broad bands, encompassing multiple transitions. In this region, we expected to see the largest difference with the salicylaldiminato series^19^ because of replacement of the substituted benzene chromophore with a substituted naphthalene chromophore in the naphthaldiminato compounds **1**–**3**. In fact, the intensities of the first two bands were enhanced by factors 1.5 and 2, respectively, and ECD spectra were also correspondingly affected. For all compounds, we observed a series of four major bands (some of which split) with a –/+/+/– sequence of signs for the *R* enantiomer, moving from the short to long wavelengths of the UV region. The most intense band has a positive maximum for *R* enantiomers around 315–318 nm. The *g* factor (Δε/ε) is ≈6 × 10^–4^ for the latter band and ≈2 × 10^–4^ for the band just below 400 nm.

The UV region is also the only region of the spectrum where it may be meaningfully compared with the homoleptic diamagnetic zinc series^26^ because here it is expected to be less affected by the transitions of the paramagnetic Co^II^ core. In fact, at least in the range between 230 and 350 nm, the two series of spectra are fairly similar, reinforcing our expectations. On the contrary, the very broad band between 350 and 450 nm was replaced by two bands of opposite sign in the zinc series, which were attributed to an exciton couplet.^26^ This is missing in the present case or obscured by other contributions; more in general, the ECD spectra of compounds **1**–**3** in the UV range do not display clear signs of exciton coupling despite the presence of the naphthaldiminato chromophores. The main reason is the following: the π–π* transition of the naphthalene ring oriented along its long axis is the most intense one and often responsible for strong exciton-coupled CD (ECCD) spectra in compounds with multiple naphthalene chromophores;^54,55^ in the current case, however, the long axes of the two naphthalene rings are almost collinear [see the density functional theory (DFT)-optimized geometries in the Supporting Information], leading to weak ECCD.

We have previously employed variable-temperature ECD measurements (VT-ECD) for investigating the diastereomeric equilibrium of cobalt(II) Schiff base metal complexes,^19^ for which NMR experiments are complicated by paramagnetic shift and peak broadening.^56,57^ In the current case, VT-ECD spectra of (*S*)-**1** measured in chloroform between −10 and +40 °C barely showed any variation above 380 nm (Figure S7), where we expected to see the effect of the equilibrium between species with different chirality at-metal.^19^ ECD data at 400 nm showed a very small increase upon temperature lowering, with an overall variation of 0.17 ± 0.1 Δε units over the measured range (50 °C). Their fitting according to a two-species equilibrium model yielded a free energy difference between the two species of ∼2 ± 0.2 kcal mol^–1^ (Figure S7). A more apparent temperature dependence of the ECD spectra was observed at shorter wavelengths (<380 nm), which cannot be interpreted as a simple two-species equilibrium (see, e.g., the absence of a common crossover point in Figure S7) and, in our interpretation based also on the calculations results (see below), may be due to equilibria involving multiple conformers of a given diastereomeric species with different ligand arrangements. In summary, VT-ECD data recorded for compound **1** demonstrated a strong preference for a single diastereomer and little, if any, hint of a solution equilibrium process with the other diastereomer.

In the *visible region* (450–800 nm), the absorption spectra display two maxima at 475 and 515 nm, plus a very broad band above 600 nm (faintly visible only for **1**). The accompanying CD spectra show two or three bands. It is known that d^7^ tetrahedral cobalt(II) complexes undergo a triply degenerate ^4^A_2_ → ^4^T_1_(P) transition around 550 nm,^58^ which, upon symmetry lowering,^59,60^ may well be responsible for the observed band pattern in **1**–**3**. In fact, a very similar sequence of bands was observed for the analogous salicylaldiminato series.^19^ The maximum *g* factor is measured for the 620 nm band of compound **1** and reaches a value of ≈2 × 10^–3^.

In the *NIR region* (800–2000 nm), we can distinguish two absorption bands around 960 and 1250 nm. We have experimental^19^ and theoretical (vide infra) evidence about the occurrence of a third transition around 1600 nm. Above 800 nm, d^7^ tetrahedral cobalt(II) complexes undergo a second triply degenerate ^4^A_2_ → ^4^T_1_(F) transition,^58^ which is again expected to be split by symmetry lowering.^59,60^ The NIR absorption profile of **1**–**3** is, in fact, very similar to the analogous salicylaldiminato series.^19^ The NIR-CD spectrum is available only up to 1100 nm with our current instrumental setup, but still we can recognize a negative tail for (*R*)-**1**–**3** and a positive one for (*S*)-**1**–**3**, which is also consistent with the salicylaldiminato series^19^ and with the calculation results (vide infra). The latest accessible *g* factor is ≈9 × 10^–3^ at 1100 nm, a fairly large value that would certainly further increase on the red edge of the NIR range.

The *IR region* (800–4000 cm^–1^) is the most interesting of the whole superspectrum because of the mixing between ligand-centered vibrational transitions and metal-centered LLESs. With our current experimental setup, we could cover the high-frequency region up to 4000 cm^–1^ in both the absorption IR and VCD components. The frequency extension further highlights the presence of a broad band clearly visible in both absorption and CD between 2400 and 4000 cm^–1^, with negative VCD for (*R*)-**1**–**3**, possibly flanked by a much weaker band of opposite sign between 1800 and 2400 cm^–1^. This band (or these bands) is (are) associated with the lowest-lying transition of the Co^II^ core, which is a triply degenerate ^4^A_2_ → ^4^T_2_ transition for tetrahedral d^7^ cobalt(II) systems observed between 2000 and 7000 cm^–1^.^58,59^ Overall, the band has a full-width at half-maximum (fwhm) of about 1000 cm^–1^, which superimposes on IR/VCD bands due to C–H stretching modes with typical fwhm values of a few reciprocal centimeters. For the electronic transition, the estimated *g* factor is remarkably around ≈2 × 10^–2^. Unfortunately, VCD bands above 3000 cm^–1^ are disturbed by a relatively large noise, so it is hard to quantify the effect of the coupling between the metal LLESs and ligand vibrations in this region, where the frequency match is at its maximum. On the other hand, the coupling affects in a spectacular way the medium-IR or fingerprint region of the VCD spectrum as well. While the absorption IR spectra are rather standard, the VCD spectra are not. Instead of showing the typical alternation of positive and negative signals, the spectra are practically monosignate: for (*R*)-**1**–**3**, almost all bands are negative, while for (*S*)-**1**–**3**, almost all bands are positive (Figures 3–5). The most significant exception above 1200 cm^–1^ is the band centered at 1537 cm^–1^, which will be discussed in detail below. Overall, the balance between the bands with dominant sign (negative for *R*) and opposite sign (positive for *R*), as judged from the ratio between the integrals of the negative and positive peaks in the range 950–1700 cm^–1^, is ≈5:1; normally one would expect a roughly 1:1 proportion (Figure 3). The unbalance is even larger for **2** and **3** (Figures 4 and 5). In addition to the almost monosignate appearance, it must be stressed too that VCD spectra of compounds **1**–**3** are also significantly intense. Many bands attain a *g* factor of ≈1 × 10^–3^, i.e., at least 1 order of magnitude larger than what is commonly observed for organic compounds in the NIR-VCD region, including similar zinc(II) and copper(II) Schiff base complexes^19,22^ and other four-coordinate metal Schiff base and related complexes.^61−64^ We have previously demonstrated that the two phenomena—monosignate appearance and enhancement—are interrelated and can both be ultimately explained as the effect of vibrational–LLES coupling.^45^ In particular, intensity enhancement and sign reversal occur when the vibrational normal modes of *B* symmetry, for complexes with effective *C*_2_ symmetry, couple with LLESs having the same *B* symmetry. As we shall see below, the same symmetry-dependent phenomenon is consistently reproduced for compounds **1**–**3**. The monosignate appearance of the VCD spectra of **1**–**3** is interrupted by the intense 1537 cm^–1^ band and is disrupted in the low-frequency region below 1200 cm^–1^, where weak bands of both signs start to appear. It should be recalled that the theories of VCD for molecules with LLES are based on a resonant effect; that is, the enhancement is maximal when the energy separation between the vibrational and excited electronic states is minimal.^29,30^ In classical terms, one might invoke a Fano-type interference mechanism with a similar energy dependence.^33^ In our interpretation, *enhancement* and *sign-reversal* occur simultaneously and are both *symmetry-dependent*; therefore, it is expected that low-frequency VCD peaks below 1200 cm^–1^, being farther in energy from the LLES occurring above 2400 cm^–1^, will be the least affected by both enhancement and sign reversal. Actually, we think that the observation of *weak* and *bisignate* VCD bands below 1200 cm^–1^ reinforces our interpretation. Because of the strong sensitivity of IR and VCD toward the specific nature of the observed species, the IR and VCD spectra of compounds **1**–**3** are very different from those of the analogous salicylaldiminato series.^19^ Additionally, there is also large variability within the series **1**–**3**, especially in some regions such as 1480–1560 and 1200–1320 cm^–1^. Thus, this is also the most useful region of the superspectrum to distinguish the current cobalt(II) complexes.

### Quantum-Mechanical Calculations

Following the same approach as that used previously for other bis(salicylaldiminato) and bis(naphthaldiminato) analogues,^19,26^ we run DFT and time-dependent DFT (TD-DFT) calculations on compound **1**, as a representative of the current series. The aims of DFT calculations were the following: (a) establishing the theoretical diastereomeric preference; (b) assigning the main bands observed in the (chiro)optical superspectrum; (c) analyzing the monosignate VCD spectrum to confirm the symmetry-dependent sign reversal and rationalizing the odd behavior of the 1537 cm^–1^ band.

The computational procedure is detailed in the Computational Section. Shortly, the X-ray structure of Λ-(*R*)-**1** (Figure 1) was used as the starting point, and the geometry for its diastereomer Δ-(*R*)-**1** was generated thereof. A conformational search was run on these two geometries with molecular mechanics, by varying all possible rotatable bonds. The various conformers were then optimized at the B3LYP/def2-SVP level in vacuo and their energies evaluated at the B3LYP-D3/def2-TZVP level using the polarizable continuum model (PCM) solvent model for chloroform (see the structures in Figure S8).^65−69^ As a result, we found that the Λ-(*R*) diastereomer was predicted to be much more stable than the Δ-(*R*) diastereomer, with the former accounting for ∼100% population at 300 K. This outcome is in agreement with the experimental evidence from VT-ECD spectra. The two most stable conformers of Λ-(*R*)-**1** differed in internal energies by 1.52 kcal mol^–1^ and accounted respectively for 92.2% and 7.2% population at 300 K. The first one was almost *C*_2_-symmetric and resembled the X-ray structure of Λ-(*R*)-**1**; the root-mean-square deviation (RMSD) between the X-ray geometry and the calculated one was 0.47 Å (Figure S9). The most stable conformer of the other isomer, Δ-(*R*)-**1**, was less stable than the Λ-(*R*) absolute minimum by 3.71 kcal mol^–1^. Finally, the two relevant conformers with the Λ-(*R*)-**1** configuration were fully optimized at the B3LYP-D3/def2-TVZP/PCM level before excited-state and frequency calculations.

**Figure 6:**
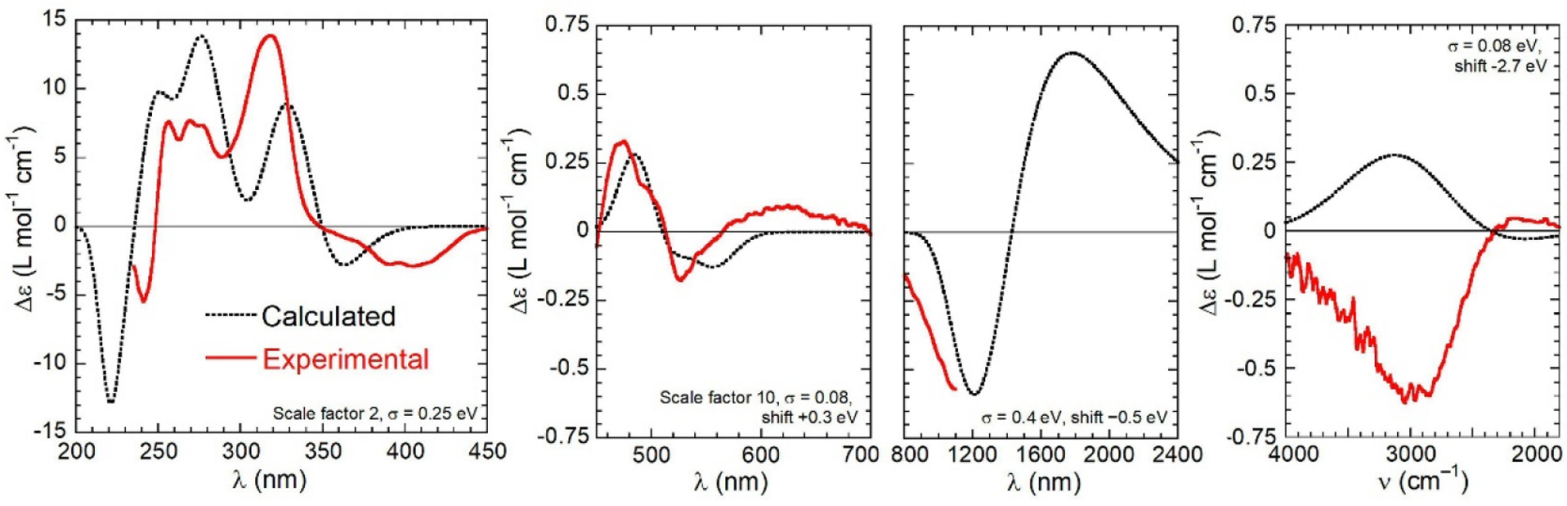
Comparison between the experimental (red lines) and calculated (black dotted lines) CD superspectra of (*R*)-**1**. Calculations run at the CAM-B3LYP/def2-TZVP//B3LYP-D3/def2-TZVP level with PCM for chloroform, as Boltzmann averages over two conformers of Λ-(*R*)-**1**. The parameters used to generate the calculated spectra in each region are given as insets.

Excited-state calculations were run with TD-DFT method on the DFT-optimized geometries of Λ-(*R*)-**1** using three functionals (B3LYP,^65,66^ CAM-B3LYP, and M06-2L^70^), the def2-TZVP basis set,^68^ and the PCM solvent model for chloroform.^69^ An exhaustive screening of the functionals, as well as a basis set assessment, had been performed previously.^19^ It must be stressed that excited-state calculations of open-shell metal complexes, especially if bound to large multichromophoric ligands, are complicated for various reasons. The most obvious one is the necessity of including dozens of excited states (roots) in the calculations to cover the whole experimentally accessible spectrum; however, it is known that TD-DFT calculations are poorly accurate for high-lying electronic transitions.^71^ Other problems arise from spin contamination, state degeneracy, spin–orbit coupling, relativistic effects, and so on.^72−75^ Still, the comparison between the experimental spectra of (*R*)-**1** recorded in chloroform and the spectra calculated as Boltzmann averages over the two populated conformers of Λ-(*R*)-**1** is generally satisfactory over a large portion of the energy range, provided that different energy and intensity corrections are applied, at both the CAM-B3LYP/def2-TZVP (Figure 6) and B3LYP/def2-TZVP (Figure S10) levels; the M06-L functional led to worse agreement (data not shown). Most ECD bands, although some of them are heavily shifted, are predicted with the correct sign; one exception is the pair of electronic bands in the IR region between 1800 and 3000 cm^–1^. The sign agreement confirms, independently from X-ray crystallography, the absolute configuration at-metal to be Λ for the *R* ligand configuration.

The calculations also substantiate the expectation that two intense ECD bands would appear in the NIR region, only one of which is experimentally accessible. Transition and Kohn–Sham orbital analyses of the electronic transitions occurring in the IR and NIR regions confirm their origin as d–d-type transitions centered on the metal; still, there is heavy mixing with ligand-centered transitions as well (Figures S11 and S12). We also verified the impact of chirality at-metal on the calculated ECD spectra by running TD-DFT calculations on the first four lowest-energy conformers found for Δ-(*R*)-**1**, which, according to B3LYP-D3/def2-TZVP/PCM//B3LYP/def2-SVP calculations, account for >86% population for the Δ-(*R*)-**1** isomer at 300 K. The Boltzmann-weighted average ECD spectrum calculated on this isomer was reversed in sign for most bands, with respect to the Λ-(*R*)-**1** isomer (Figure S13), over the whole calculated range. This finding corroborates the presence of a dominant Λ-(*R*) diastereomer in solution and demonstrates again that for the current series of compounds **1**–**3** the chiroptical response is dominated by, and it immediately reports, the chirality at-metal. It is noteworthy that the very same behavior has been consistently demonstrated for the Schiff base complexes of several metals (Co, Zn, Ni, and Cu), spanning different coordination geometries, as well as for both the salicylaldiminato and naphthaldiminato ligands.^19−21,23,26^

**Figure 7:**
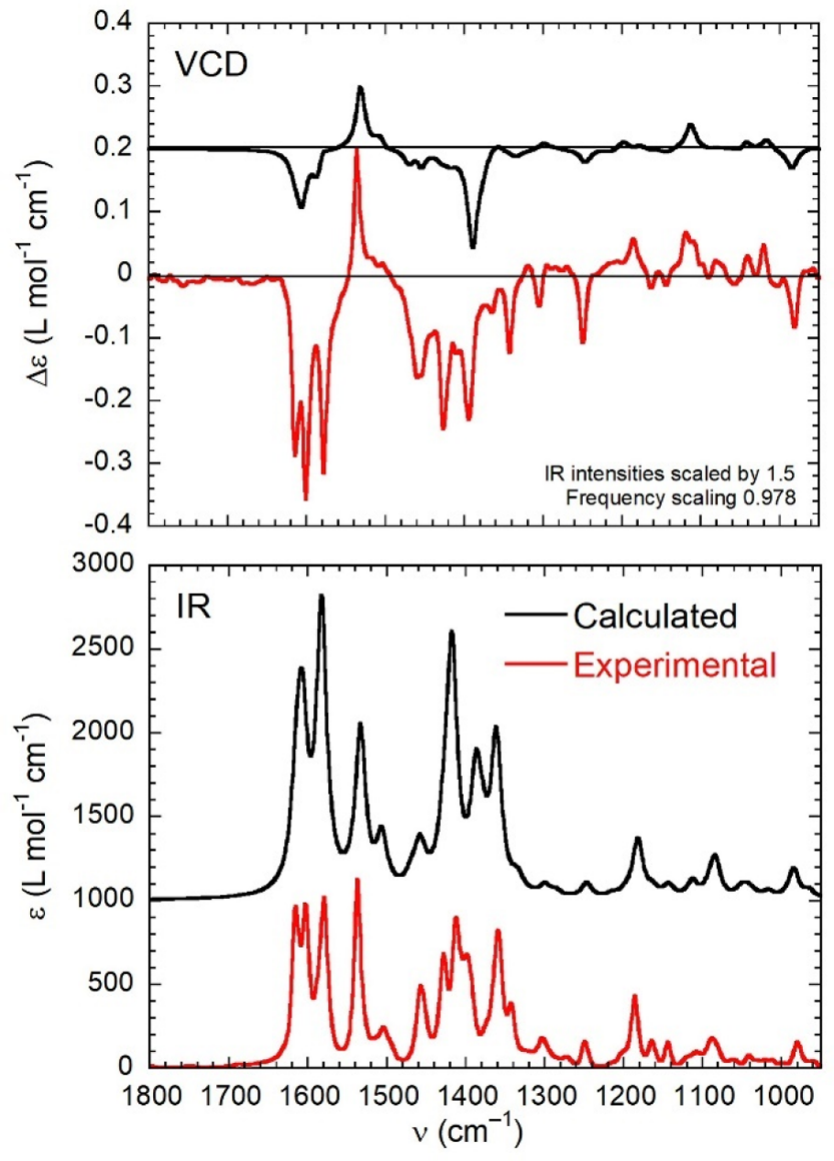
Comparison between the experimental (red lines) and calculated (black lines) VCD (top) and IR (bottom) spectra of (*R*)-**1**. Calculations run at the B3LYP/def2-TZVP level with PCM for chloroform on the *C*_2_-symmetric structure of Λ-(*R*)-**1**. The parameters used to generate the calculated spectra are given in the inset.

The simulation of the IR region of the superspectrum was pursued by running frequency calculations on the lowest-energy structure of Λ-(*R*)-**1** after reoptimization of its geometry imposing *C*_2_-symmetry restraint. The justification for this simplification is 3-fold: (a) the almost *C*_2_-symmetric conformer was by far the most stable at any level of calculation with B3LYP-D3; (b) the deviation between the symmetry-unrestricted and -restricted structures was negligible; (c) we wanted to classify the normal modes according to their *A* or *B* symmetry in the molecular *C*_2_ group, to confirm our previous finding about the symmetry dependence of the vibrational–LLES coupling.^45^ First, we verified that the first three LLESs calculated at the TD-CAM-B3LYP/def2-TZVP and TD-B3LYP/def2-TZVP levels (PCM for chloroform) all had *B* symmetry (Figure S12), which is essential for the symmetry-dependent coupling.^45^ Then, we compared the experimental and calculated absorption IR and VCD spectra in the medium-IR region (Figure 7), finding that the absorption IR spectrum is well reproduced by the calculation, which also supports our choice of the input structure. Conversely, the VCD spectrum is poorly reproduced in several aspects: many bands, including several intense negative bands, are missing; the *g* value is underestimated by a factor 10 or more; although there is an unbalance between the negative and positive bands, the integral ratio in the range 950–1700 cm^–1^ is ≈2.4:1, i.e., half that of the experiment. The poor performance of the calculations is due to the fact that the current implementation of VCD calculations in Gaussian software cannot take into account the effects of LLES, being limited to molecules with well-separated ground and electronic excited states.^29−31,35^ A necessary prerequisite for a correct treatment of vibrational–LLES coupling requires one to overcome the Born–Oppenheimer (BO) approximation; that is, it would require one to keep the electronic–vibrational coupling terms in the Hamiltonian.^29^ Recently, Tomeček and Bouř calculated the VCD spectra of our Schiff base salicylaldiminatocobalt(II) complex (analogue of **1**) going beyond the BO limit, and although they could reproduce many features of the experimental spectrum, including, e.g., the intensity enhancement, its monosignate appearance was not captured by the calculations.^76^ Thus, other ingredients of the recipe seem to be missing for a complete simulation of the VCD spectra of these systems.

**Figure 8:**
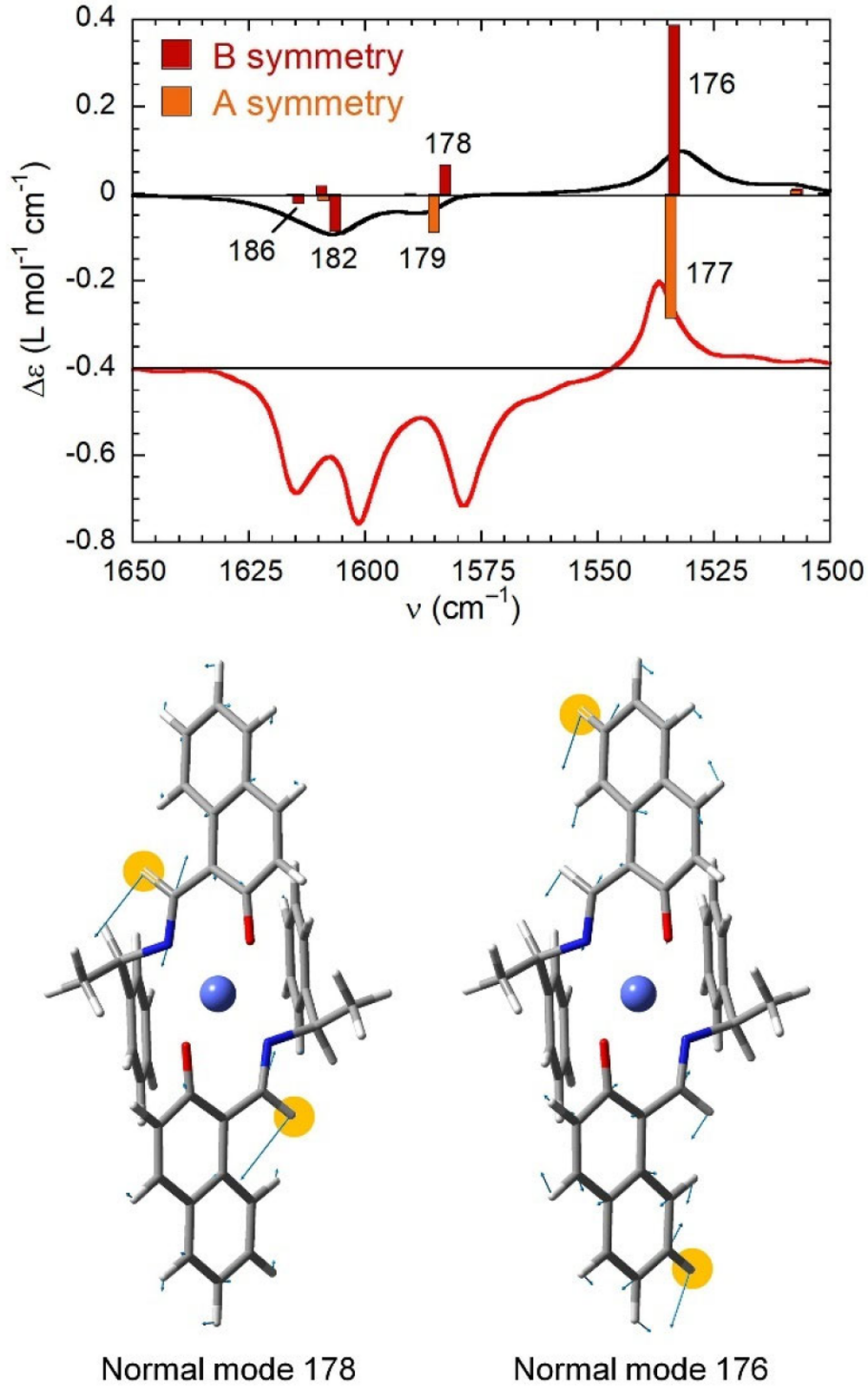
Top: Calculated VCD spectrum (black line) in the 1500–1650 cm^–1^ range for the *C*_2_-symmetric structure of (*R*)-**1** with band assignment, compared with the experimental spectrum (red line). The plotted parameters are the same as those in Figure 7. Bottom: Atom displacements (light-blue arrows) for *B*-symmetry normal modes 176 and 178. The H atoms contributing most to the normal modes are highlighted.

We analyzed more in detail the region of the VCD spectrum of (*R*)-**1** comprised between 1520 and 1620 cm^–1^. In the experimental VCD spectrum, there are four major bands, three of which are negative at 1580, 1600, and 1615 cm^–1^ and one of which is positive at 1537 cm^–1^, by far the most intense positive VCD band of the whole spectrum. The band at 1580 cm^–1^ is due to normal modes 178 and 179, respectively, with *B* and *A* symmetry, the former of which is apparently subjected to symmetry-dependent sign reversal due to coupling with *B*-symmetric LLES, judging from the comparison of the experimental and calculated spectra (Figure 8). The band at 1537 cm^–1^ is due to normal modes 176 and 177, again respectively with *B* and *A* symmetry, the former of which is not subjected to sign reversal, despite having the correct *B* symmetry (Figure 8). An explanation is thus due for the different behaviors of normal modes 176 and 178. Mode 178 is allied with the antisymmetric combination of in-plane bending vibrations of the imine C–H bonds, which lie very close to the Co^II^ core (the distance between the involved H and Co atoms is 3.9 Å in the DFT-optimized structure). Mode 176 is allied with a more composite combination of local motions, most being in-plane bending vibrations of aromatic C–H bonds localized on the naphthalene rings (see the normal mode in Figure 8); in particular, the strongest contribution comes from naphthyl C–H7, which is quite far from the Co^II^ core (the H···Co distance is 7.7 Å). We propose here that the different behavior of the two selected normal modes, which are very close in energy, is due to the different positions of the contributing bonds with respect to the Co^II^ core, which accounts for a different effect of vibrational–LLES coupling on the VCD bands: the larger the distance, the smaller the effect. Concerning the two remaining negative bands in the investigated region, the 1600 cm^–1^ band is allied with the *B*-symmetric normal mode 182, and the 1615 cm^–1^ band is allied with the *B*-symmetric normal mode 186; we believe that this latter mode is enhanced by the same symmetry-dependent coupling mechanism, being associated with in-plane bending vibrations, which involve several aromatic C–H bonds including naphthyl H8 (H···Co distances of 5.7 Å; see the normal modes in Figure S14). Domingos et al. have previously reported a distance-dependent VCD amplification effect for amino acids, dipeptides, and tripeptides complexed with the Co^2+^ ion in D_2_O.^41^ The same authors also reported enhanced VCD spectra for a ferrocenium-derivatized oligopeptide and demonstrated how the amplification decreases exponentially with the distance, lending itself as a spectroscopic ruler.^77^ In all cases, the enhanced VCD spectra consisted of positive and negative bands. The same distance-dependent effect seems to be at play here; however, this is the first time that the *distance* and *symmetry* dependences of vibrational–LLES coupling are observed together, in relation to the *monosignate* VCD appearance and sign reversal. We think that the method for visualizing the vibrational transition current densities developed by Fusè et al.^78^ might help in the interpretation of the phenomena observed for the two analogous series of cobalt(II) complexes, possibly highlighting both their distance and symmetry dependences.

VCD enhancement theories embed a distance dependence in the mixing between the vibrational and electronic wave functions, although this dependence is not made explicit like, for example, the off-resonance effect (vide supra).^29,30^ On the other hand, VCD enhancement can be classically interpreted as due to the combination between an electric-dipole-allowed state—the vibrational one—and a magnetic-dipole-allowed state—the LLES one.^79,80^ In this mechanism, called the dynamic or μ–*m* coupling mechanism for optical activity, the rotational strength *R*_μ*m*_ arises from the first-order mixing between the two states with respectively nonzero electric μ and magnetic *m* transition dipoles, mediated by a coupling potential *V*_μ*Q*_ (*Q* is the electric quadrupole associated with *m*).^59^ The mixing is explicitly subjected to (a) a resonance criterion, with *R*_μ*m*_ being inversely proportional to the difference of the squares of the state energies; (b) symmetry rules for a constructive interaction between transition densities (described through *V*_μ*Q*_), which have been historically simplified in terms of sector rules;^81^ (c) a well-defined distance dependence, with *R*_μ*m*_ being proportional to the inverse fourth or sixth power (depending on the nature of the LLES involved) of the distance between the two dipoles.^79,80^ It is interesting to notice how the same rules seem to consistently hold for symmetry- and distance-dependent VCD enhancement in the presence of LLES.

## Conclusions

With the present paper, we further extended the family of metal(II) Schiff base complexes **1**–**3** exhibiting distorted square-planar geometries and chirality at-metal. We reported here the naphthaldiminatocobalt(II) series, which compares, on the one hand, to the salicylaldiminatocobalt(II) analogues^19^ and, on the other hand, with the homoleptic zinc(II),^26^ copper(II),^20^ and nickel(II)^21^ series. Similar to all other mentioned analogues, a complete diastereoselectivity is suggested in the solid crystalline state, meaning that, for a certain ligand absolute configuration, only one metal configuration is obtained selectively in the investigated single crystal, e.g., Λ-(*R*) and Δ-(*S*). DSC analyses revealed a single-phase transformation from the crystalline to the isotropic liquid state for **1**–**3**. Complexes **1**–**3** were characterized by broad-range chiroptical spectroscopies extending from the UV to medium-IR region of the spectrum. The effect of the presence of the high-spin d^7^ Co^2+^ core is especially evident in the NIR and IR regions (800–1200 nm and 2000–4000 cm^–1^, respectively) but also has a dramatic impact on the fingerprint IR region (900–1800 cm^–1^), thanks to the coupling between the ligand vibrations and metal-centered LLESs. The medium-IR VCD spectrum is almost monosignate and enhanced in intensity. We had demonstrated before that the two phenomena are interrelated and also symmetry-dependent for *C*_2_-symmetric molecules.^45^ Here, we show that they are also distance-dependent, namely, vibrational–LLES coupling operates more strongly for normal modes closer to the Co^2+^ core. This latter phenomenon had also been demonstrated before^30^ but not in conjunction with the symmetry dependence. Thus, it is the first time that the *distance* and *symmetry* dependences of vibrational–LLES coupling are observed together, in relation to the *monosignate* VCD appearance and sign reversal. We also noticed how the experimental observations might be interpreted in the framework of the “classical” dynamic or μ–*m* coupling mechanism for optical activity. Finally, our experimental and computational evidence does not highlight for compounds **1**–**3** any diastereomeric equilibrium between, e.g., Λ-(*R*) and Δ-(*R*) species, in solution, where a single isomer Λ-(*R*) prevails. This is at odds with many analogous Schiff base complexes for which the equilibrium was observed before,^19,20,26^ demonstrating that a proper choice of the ligand and/or metal is a means to modulating the dynamic properties of the complexes in solution.

## Materials and Methods

Cobalt(II) acetate tetrahydrate and NaHCO_3_ were used as received from Merck (Germany). Commercial-grade methanol, distilled over CaCl_2_, was used for synthesis of the complexes. IR (attenuated total reflectance, ATR) spectra were recorded on a Nicolet iS10 spectrometer at ambient temperature. Elemental analyses were run on a Vario EL instrument from Elementar Analysensysteme. EI-MS spectra were acquired with a Thermo-Finnigan TSQ 700 spectrometer. Isotopic distribution patterns for ^58/60^Co [in Co-(*R* or *S*)-L1 and Co-(*R* or *S*)-L2] or combined ^58/60^Co + ^79/81^Br [Co-(*R* or *S*)-L3] containing ions are detected in the mass spectra. DSC was run on a Shimadzu DSC-60A heat-flux instrument (working in endodown mode) in the range 30–240 °C (just up to decomposition temperature) with a rate of 10 K min^–1^ under a nitrogen atmosphere. NMR spectra were measured on a Bruker Avance III-300 spectrometer.

### (Chiro)optical Superspectra

*ECD and UV–vis* spectra were recorded with a Jasco J-715 spectropolarimeter (Tokyo, Japan) in the range of 230–800 nm at room temperature in spectroscopic-grade CHCl_3_ (∼0.2 mM) in quartz cells with path lengths of 0.1 cm (UV region) and 2 cm (visible region). All spectra were measured using a scanning speed of 100 nm min^–1^, a step size of 0.2 nm, a bandwidth of 2 nm, a response time of 0.5 s, and an accumulation of four scans. The spectra were background-corrected using spectra of CHCl_3_ recorded under the same conditions.

*NIR-CD* spectra were recorded with a Jasco J-815 spectropolarimeter (Tokyo, Japan) equipped with an extended wavelength detector in the range of 800–1100 nm at room temperature in spectroscopic-grade CHCl_3_ (∼0.8 mM) in a quartz cell with a path length of 2 cm. All spectra were measured using a scanning speed of 200 nm min^–1^, a step size of 0.5 nm, a bandwidth of 10 nm, a response time of 0.5 s, and an accumulation of four scans. The spectra were background-corrected using spectra of CHCl_3_ recorded under the same conditions.

*NIR absorption* spectra were recorded using a Jasco V-670 spectrophotometer (Tokyo, Japan) in the range of 800–2700 nm at room temperature in spectroscopic-grade CHCl_3_ (∼0.8 mM) in a quartz cell with a path length of 2 cm. All spectra were measured using a scanning speed of 200 nm min^–1^, a step size of 0.5 nm, a bandwidth of 8 nm, and a fast response time. The spectra were background-corrected using spectra of CHCl_3_ recorded under the same conditions.

*VCD and IR* spectra in the subrange 2000–900 cm^–1^ were recorded at a resolution of 4 cm^–1^ for ∼40 mM solutions in CDCl_3_ at room temperature using a Jasco FVS-6000 spectrometer (Tokyo, Japan). In the subrange 4000–2000 cm^–1^, VCD and IR spectra at the same resolution were recorded for ∼50 mM solutions in CDCl_3_ at room temperature using a BioTools Chiral *IR*-2X spectrometer (Jupiter, USA). All solutions were measured in BaF_2_ cells with a path length of 200 μm for 40 min (2000–950 cm^–1^) and 100 μm for 10 h (4000–2000 cm^–1^) to improve the signal-to-noise ratio. The optimum retardation value of the photoelastic modulator (PEM) was set at 1400 cm^–1^ for 2000–950 cm^–1^ and at 2800 cm^–1^ for 4000–2000 cm^–1^. The spectra were averaged and background-corrected using spectra of CDCl_3_ recorded under the same conditions. The average VCD noise level was ±0.05 to ±0.10 dm^3^ mol^–1^ cm^–1^ in the range 4000–2000 cm^–1^ and ±0.005 dm^3^ mol^–1^ cm^–1^ in the range 2000–950 cm^–1^.

### General Procedure for the Synthesis of Napthaldiminatocobalt(II) Complexes

Enantiopure Schiff base (*R* or *S*)-*N*-1-(phenyl)ethyl-2-hydroxy-1-naphthaldimine [(*R*)/(*S*)-HL1] (137.6 mg, 0.5 mmol) was dissolved in 5 mL of methanol and stirred for ca. 5 min. NaHCO_3_ (42 mg, 0.5 mmol) dissolved in 5 mL of hot methanol was added to the Schiff base solution and stirred for ca. 10 min. Afterward, cobalt(II) acetate tetrahydrate (62.3 mg, 0.25 mmol) dissolved in 5 mL of methanol was added to the deprotonated Schiff base solution and continued to be stirred at 60–70 °C for ca. 6 h under nitrogen. The color changed from light red to deep pink-red. The solution was filtered off, and the volume of the solvent was reduced to ca. 50% in vacuo. This concentrated solution was left standing for crystallization via slow evaporation of the solvent at room temperature. The deep-pink-red crystals of (*R*)-**1** or (*S*)-**1**, suitable for X-ray measurements, were obtained within 4–5 days. The crystals were separated, washed with methanol followed by *n*-hexane (0.5 mL in each), and dried in vacuo. The complexes (*R*)-**2** or (*S*)-**2** and (*R*)-**3** or (*S*)-**3** were synthesized following the same procedure using (*R* or *S*)-*N*-1-(*p*-methoxyphenyl)ethyl-2-hydroxy-1-naphthaldimine [(*R*)/(*S*)-HL2] and (*R* or *S*)-*N*-1-(*p*-bromophenyl)ethyl-2-hydroxy-1-naphthaldimine [(*R*)/(*S*)-HL3], respectively.

#### Bis[(*R* or *S*)-*N*-1-(phenyl)ethyl-2-oxo-1-naphthaldiminato-κ^2^*N*,*O*]cobalt(II), (*R*)-**1** or (*S*)-**1**

Yield: 110 mg (72%). IR (KBr, cm^–1^): 3055, 3032, 2967, 2928w (H–C), 1616, 1601vs (C=N), 1578vs (C=C). EI-MS for (*R*)-**1**: *m*/*z* 607 (80%, [M]^+^), 502 (28%, [C_10_H_6_(CHNH)(O)CoL1 – H]^+^), 488 (27%, [C_10_H_6_(CHNH)(O)CoL1 + H]^+^), 332 (100%, [CoL1 – H]^+^), 275 (14%, [HL1]^+^), 257 (42%, [C_10_H_6_(O)(CHNH(CH_2_CH_3_)Co – H]^+^), 229 (99%, [C_10_H_6_(CHNH)(O)Co]^+^), 105 (50%, [(C_6_H_5_)(CH_3_)CH]^+^). EI-MS for (*S*)-**1**: *m*/*z* 607 (12%, [M]^+^), 502 (8%, [C_10_H_6_(CHNH)(O)CoL1 – H]^+^), 488 (5%, [C_10_H_6_(CHNH)CoL1 + H]^+^), 332 (63%, [CoL1 – H]^+^), 275 (17%, [HL1]^+^), 257 (22%, [C_10_H_6_(O)(CHNH(CH_2_CH_3_)Co – H]^+^), 229 (100%, [C_10_H_6_(CHNH)(O)Co]^+^), 105 (90%, [(C_6_H_5_)(CH_3_)CH]^+^) (M = C_38_H_32_CoN_2_O_2_; HL1 = C_19_H_17_NO). Anal. Calcd for C_38_H_32_N_2_O_2_Co (607.62): C, 75.12; H, 5.31; N, 4.61. Found for (*R*)-**1**: C, 75.16; H, 5.21; N, 4.55. Found for (*S*)-**1**: C, 74.85; H, 5.44; N, 4.46.

#### Bis[(*R*)-*N*-1-(*p*-methoxyphenyl)ethyl-2-oxo-1-naphthaldiminato-κ^2^*N*,*O*]cobalt(II), (*R*)-**2** or (*S*)-**2**

Yield: 120 mg (72%). IR (ATR, cm^–1^): 3051, 2990, 2933w (H–C), 1614, 1601vs (C=N), 1582vs (C=C). EI-MS for (*R*)-**2**: *m*/*z* 667 (5%, [M]^+^), 532 (8%, [C_10_H_6_(CHNH)(O)CoL2 – H]^+^), 362 (10%, [CoL2 – H]^+^), 305 (5%, [HL2]^+^), 229 (11%, [C_10_H_6_(CHNH)(O)Co]^+^), 135 (100%, [(CH_3_)(C_6_H_4_OCH_3_)CH]^+^), 105 (12%, [(C_6_H_5_)(CH_3_)CH]^+^). EI-MS for (*S*)-**2**: *m*/*z* 667 (16%, [M]^+^), 532 (20%, [C_10_H_6_(CHNH)(O)CoL2 – H]^+^), 362 (10%, [CoL2 – H]^+^), 305 (5%, [HL2]^+^), 229 (15%, [C_10_H_6_(CHNH)(O)Co]^+^), 135 (100%, [(CH_3_)(C_6_H_4_OCH_3_)CH]^+^), 105 (15%, [(C_6_H_5_)(CH_3_)CH]^+^) (M = C_40_H_36_N_2_O_4_Co; HL2 = C_20_H_18_NO_2_H). Anal. Calcd for C_40_H_36_N_2_O_4_Co (667.67): C, 71.96; H, 5.44; N, 4.20. Found for (*R*)-**2**: C, 71.43; H, 5.30; N, 4.09. Found for (*S*)-**2**: C, 71.97; H, 5.78; N, 4.68.

#### Bis[(*R* or *S*)-*N*-1-(*p*-bromophenyl)ethyl-2-oxo-1-naphthaldiminato-κ^2^*N*,*O*]cobalt(II), (*R*)-**3** or (*S*)-**3**

Yield: 130 mg (68%). IR (ATR, cm^–1^): 3054, 2980, 2930w (H–C), 1614, 1598vs (C=N), 1579vs (C=C). EI-MS for (*R*)-**3**: *m*/*z* 765 (10%, [M]^+^), 582 (10%, [M – (C_6_H_4_Br)(CH_3_)CH]^+^), 410 (21%, [CoL3 – H]^+^), 353 (12%, [HL3]^+^), 332 (22%, [C_10_H_6_(CHNH)(CHCH_3_C_6_H_4_(O)Co – H]^+^), 273 (100%, [HL3 – HBr]^+^), 256 (15%, [C_10_H_6_(O)(CHNH(CHCH_3_)Co – H]^+^), 229 (90%, [C_10_H_6_(O)(CHNH)Co + H]^+^), 183 (30%, [(C_6_H_4_Br)CH_3_)CH]^+^), 170 (35%, [C_10_H_6_(O)(NH)CH]^+^), 104 (85%, [(C_6_H_5_)(CH_3_)CH – H]^+^) (isotopic distributions patterns resulting from ^79/81^Br containing ions are clearly visible following the peaks at *m*/*z* 765, 582, 410, and 183, respectively). EI-MS for (*S*)-**3**: 765 (55%, [M]^+^), 582 (34%, [M – (C_6_H_4_Br)(CH_3_)CH]^+^), 410 (32%, [CoL3 – H]^+^), 353 (15%, [HL3]^+^), 332 (22%, [C_10_H_6_(CHNH)(CHCH_3_C_6_H_4_(O)Co – H]^+^), 273 (79%, [HL3 – HBr]^+^), 256 (21%, [C_10_H_6_(O)(CHNH(CHCH_3_)Co – H]^+^), 229 (100%, [C_10_H_6_(O)(CHNH)Co]^+^), 183 (35%, [(C_6_H_4_Br)CH_3_)CH]^+^), 170 (42%, [C_10_H_6_(O)(NH)CH]^+^), 104 (85%, [(C_6_H_5_)(CH_3_)CH – H]^+^) (M = C_38_H_30_Br_2_CoN_2_O_2_; HL3 = C_19_H_15_BrNOH). Calcd for C_38_H_30_N_2_O_2_Br_2_Co (765.43): C, 59.63; H, 3.95; N, 3.66. Found for (*R*)-**3**: C, 59.24; H, 3.62; N, 3.42. Found for (*S*)-**3**: C, 59.68; H, 3.95; N, 3.67.

### X-ray Crystallography

Single crystals of Λ-Co-(*R*)-L1, Δ-Co-(*S*)-L1, Λ-Co-(*R*)-L2, and Δ-Co-(*S*)-L3 were carefully selected under a polarizing microscope and mounted on a loop. *Data collection*: Bruker APEX II CCD diffractometer with graphite- or multilayer-mirror-monochromated Mo Kα radiation (λ = 0.71073 Å); ω scans. Data collection and cell refinement with *APEX2*(82) data reduction with *SAINT* (Bruker).^83^*Structure analysis and refinement*: The structures were solved by direct methods (*SHELXT-2015*),^83^ refinement was done by full-matrix least squares on *F*^2^ using the *SHELXL-2017/1* program suite, empirical (multiscan) absorption correction with *SADABS* (Bruker).^84,85^ All non-H positions were refined with anisotropic temperature factors. H atoms for aromatic and olefinic CH, aliphatic CH, and CH_3_ groups were positioned geometrically (C–H = 0.94 Å for aromatic and olefinic CH, 0.99 Å for aliphatic CH, and 0.97 Å for CH_3_) and refined using a riding model (AFIX 43 for aromatic/olefinic CH, AFIX 13 for aliphatic CH, and AFIX 137 for CH_3_), with *U*_iso_(H) = 1.2*U*_eq_(CH) and *U*_iso_(H) = 1.5*U*_eq_(CH_3_). Details of the X-ray structure determinations and refinements are provided in Table S1. Graphics were drawn with *Diamond* (version 4.4).^86^

### Computational Section

Conformational searches and preliminary DFT calculations were run with *Spartan18* (Irvine, CA, 2019) using default grids and convergence criteria. DFT and TD-DFT calculations were run with the *Gaussian16* suite^87^ using default grids and convergence criteria. The X-ray structure of Λ-(*R*)-**1** was used as the starting geometry for the calculations and to generate a starting geometry of Δ-(*R*)-**1**. In the first step, a conformational search was run on both Λ-(*R*)-**1** and Δ-(*R*)-**1** with molecular mechanics (Merck Molecular force field, MMFF) using the Monte Carlo algorithm implemented in *Spartan18*. All structures thus found were preoptimized with DFT at the B3LYP/6-31G(d) level in *Spartan18* and then at the B3LYP/def2-SVP level in vacuo in *Gaussian16*. The procedure yielded four conformers for Λ-(*R*)-**1** and eight conformers for Δ-(*R*)-**1** within 10 kcal mol^–1^ (Figure S8). All conformers were reevaluated at the B3LYP-D3/def2-TZVP/PCM level using the IEF-PCM solvent model (for CHCl_3_), and the two most stable conformers for Λ-(*R*)-**1** were fully reoptimized at the same level before the next steps.

TD-DFT calculations were run on all relevant DFT minima with the B3LYP, CAM-B3LYP, and M06-L functionals, in combination with the def2-TZVP basis set (on all atoms) and the PCM solvent model for chloroform. The calculations included from 60 to 80 excited states (roots). Spectra were averaged according to the Boltzmann distribution at 300 K. Frequency calculations (providing IR and VCD spectra) were run at the B3LYP/def2-TZVP level using PCM for CHCl_3_.

The absorption, ECD, and VCD spectra were averaged and plotted using the software *SpecDis* (version 1.71).^88,89^ Kohn–Sham orbitals and transition densities were plotted with the software *MultiWfn* (version 3.4).^90^

## Supporting Information Available

Additional characterization, CD, and computational data (PDF)

## Accession Codes

CCDC 2083905–2083908 contain the supplementary crystallographic data for this paper. These data can be obtained free of charge via www.ccdc.cam.ac.uk/data_request/cif, or by emailing data_request@ccdc.cam.ac.uk, or by contacting The Cambridge Crystallographic Data Centre, 12 Union Road, Cambridge CB2 1EZ, UK; fax: +44 1223 336033.
