# Supplementary material for: Synthesis and Characterization of Bis[(R or S)-N-1-(X-C6H4)ethyl-2-oxo-1-naphthaldiminato-κ2N,O]-Λ/Δ-cobalt(II) (X = H, p-CH3O, p-Br) with Symmetry- and Distance-Dependent Vibrational Circular Dichroism Enhancement and Sign Inversion
Source: Inorg Chem. 2021 Sep 3;60(18):14116–31. doi: 10.1021/acs.inorgchem.1c01503 (PMC8456410; doi:10.1021/acs.inorgchem.1c01503)
Supplement: Supplementary file 1 — ic1c01503_si_001.pdf [file ic1c01503_si_001.pdf]

Supporting Information

Synthesis and characterization of *bis*[(*R* or *S*)-*N*-1-(X-C_6H_4)ethyl-2-oxo-1-naphthaldiminato-κ^2*N,O*]-Λ/Δ-cobalt(II) (X = H, *p*-CH_3O, *p*-Br) with symmetry- and distance-dependent VCD enhancement and sign inversion

Marcin Górecki,[†,‡] *Mohammed Enamullah,*[⊥,*] *Mohammad Ariful Islam,*[⊥] *Mohammad Khairul Islam,*[⊥] *Simon-Patrick Höfert,*[‖] *Dennis Woschko,*[‖] *Christoph Janiak,*[‖,*] *and Gennaro Pescitelli*[†,*]

[†] Department of Chemistry and Industrial Chemistry, University of Pisa, Pisa, Italy. E-mail: gennaro.pescitelli@unipi.it (G.P.)

[‡] Institute of Organic Chemistry, Polish Academy of Sciences, Warsaw, Poland.

[⊥] Department of Chemistry, Jahangirnagar University, Dhaka, Bangladesh. E-mail: enamullah@juniv.edu (M.E.)

[‖] Institute of Inorganic Chemistry and Structural Chemistry, Heinrich-Heine-University of Düsseldorf, Düsseldorf, Germany. E-mail: janiak@uni-duesseldorf.de (C.J.)

Contents

Table S1. Crystal data and structure refinements for Co(II)-Schiff bases complexes. S3

Table S2. Selected bond lengths (Å) and angles (°) in Δ-Co-*S*-L1 [(*S*)-**1**] and Λ-Co-*R*-L1 [(*S*)-**1**] . S4

Table S3. Selected bond lengths (Å) and angles (°) in Λ-Co-*R*-L2 [(*R*)-**2**] and Δ-Co-S-L3 [(*S*)-**3**]. S4

Table S4. Ligand chirality and substituents leading to chirality induction at-metal in *bis*[(*R*- or *S*)-N-1-(*p*-X-C_6H_4)ethyl-salicylaldiminato-k^2*N*,*O*]-Λ- or Δ-M(II) in the solid state and in solution..... S5

Figure S1. EI-mass spectra for (a) Co-*R*-L1 [(*S*)-**1**] and Co-*S*-L1 [(*S*)-**1**]; (b) Co-*R*-L2 [(*R*)-**2**] and Co-*R*-L2 [(*R*)-**2**]; (c) Co-*R*-L3 [(*R*)-**3**] and Co-*S*-L3 [(*S*)-**3**] S6

Figure S2. X-ray molecular structures of Δ-Co-*S*-L**1** [(*S*)-**1**]... S9

Figure S3. X-ray molecular structures of Λ-Co-*R*-L**1** [(*R*)-**1**] S10

Figure S4. X-ray molecular structure in Λ-Co-*R*-L**2** [(*R*)-**2**] S11

Figure S5. X-ray molecular structures of Δ-Co-*S*-L**3** [(*S*)-**3**].. S12

Figure S6. Differential Scanning Calorimetry (DSC) curves for complexes (*R*)-**1**, (*S*)-**1**, (*R*)-**2**, and (*S*)-**2**, and ligand HL2.. S13

Figure S7. VT-ECD spectra of (*S*)-**1** in $CHCl_3$ and data fitting.. S14

Figure S8. Geometries and relative energies calculated for (*R*)-**1** with DFT................................. S15

Figure S9. Molecular overlay between the X-ray structure of (*R*)-**1** and the lowest-energy DFT-optimized structure... S16

Figure S10. Comparison of experimental and B3LYP-calculated CD superspectra of (*R*)-**1** S17

Figure S11. Kohn-Sham MO plots and energies calculated for (*R*)-**1** S18

Figure S12. Transition energies, symmetries and density plots of (*R*)-**1** S19

Figure S13. Comparison of calculated ECD spectra for Λ-(*R*)-**1** and Δ-(*R*)-**1** S20

Figure S14. Atom displacements for B-symmetry normal modes 182 and 186 .... S21

References for the Supporting Information S22

Table S1. Crystal data and structure refinements for Co(II)-Schiff bases complexes.

	Λ-Co-*R*-L1 [(*R*)-**1**]	Δ-Co-*S*-L1 [(*S*)-**1**]	Λ-Co-*R*-L2 [(*R*)-**2**]	Δ-Co-*S*-L3 [(*S*)-**3**]
Empirical formula	$C_{38}H_{32}CoN_2O_2$	$C_{38}H_{32}CoN_2O_2$	$C_{40}H_{36}CoN_2O_4$	$C_{38}H_{30}Br_2CoN_2O_2$
M (g mol^{-1})	607.58	607.58	667.64	7.65.39
Temperature (K)	140	140	140	140
Crystal size	0.372 × 0.211 × 0.166	0.125× 0.125 × 0.05	0.06 × 0.02 × 0.01	0.312 × 0.254 × 0.214
Crystal system	Triclinic	Triclinic	Monoclinic	Trigonal
Space group	P1	P1	$P2_1$	$P3_221$
a (Å)	9.4050(6)	9.4128(8)	8.8350 (6)	10.1323(4)
b (Å)	9.4342(6)	9.4440(8)	19.1145 (11)	10.1323(4)
c (Å)	18.7375(11)	18.7617(15)	10.2002 (6)	54.390(2)
α (°)	80.145(4)	80.190(4)	90	90
β (°)	88.372(4)	88.490(4)	96.594 (3)	90
γ (°)	64.184(3)	64.170(3)	90	120
V(Å^3)	1472.55(16)	1477.1(2)	1711.18 (18)	4835.8(4)
$Z/D_{calc.}$(g cm^{-3})	2/1.370	2/1.366	2/1.296	6/1.577
μ (mm^{-1})	0.621	0.619	0.545	3.049
F(000)	634	634	698	2310
Max./min. transmission	1.000/0.9195	1.000/0.947	1.000/0.915	1.000/0.807
θ range (°)	2.209-25.599	2.206-27.769	2.010-27.599	2.439-26.855
h; k; l ranges	±11; ±11; ±22	±12; ±12; ±24	-11, 9; ±24; -12, 13	-11, 12; -12, 10; ±69
Reflections collected	20958	57134	27042	24760
Independent reflect. (R_{int})	10575 (0.027)	13750 (0.030)	7660 (0.035)	6801 (0.049)
Data completeness (%)	95.0	99.2	97.9	99.2
Data/restraints/parameters	10575/3/778	13750/3/774	7660/1/428	6801/0/409
Goodness-of-fit on F^2[a]	1.022	1.031	0.795	1.090
R_1/wR_2 [I>2σ (I)][b]	0.0347/0.0803	0.0286/0.0703	0.0375/0.1005	0.0468/0.1065
R_1/wR_2 (all data)[b]	0.0395/0.0833	0.0318/0.0718	0.0516/0.1134	0.0544/0.1109
Max./min. $\Delta\rho$ (e. Å^{-3})[c]	0.40/-0.28	0.598/-0.222	0.264/-0.292	0.649/-0.395
Flack parameter[d]	0.005(6)	0.007(9)	0.014(5)	0.050(6)
CCDC number	2083905	2083906	2083907	2083908

[a] Goodness-of-fit = $[\sum[w(F_o^2 - F_c^2)^2]/(n - p)]^{1/2}$; [b] $R_1=[\sum(||F_o| - |F_c||)/\sum|F_o|]$; $wR_2=[\sum[w(F_o^2 - F_c^2)^2]/\sum[w(F_o^2)^2]]^{1/2}$;

[c] Largest difference peak and hole; [d] Absolute structure (Flack) parameter.[1-4]

Table S2. Selected bond lengths (Å) and angles (°) in Δ-Co-*S*-L1 [(*S*)-**1**] (A and B)[a] and Λ-Co-*R*-L1 [(*R*)-**1**] (A and B).[a]

Δ-Co-*S*-L1 (A)		Δ-Co-*S*-L1 (B)		Λ-Co-*R*-L1 (A)		Λ-Co-*R*-L1 (B)	
Co1-O1/O2	1.916(2)/1.917(2)	Co2-O3/O4	1.910(2)/1.913(2)	Co1-O1/O2	1.912(3)/1.916(3)	Co2-O3/O4	1.907(3)/1.912(3)
Co1-N1/N2	1.976(3)/1.971(2)	Co2-N3/N4	1.969(3)/1.972(2)	Co1-N1/N2	1.973(3)/1.977(3)	Co2-N3/N4	1.967(4)/1.967(3)
O1-C1/O2-C20	1.297(3)/1.309(3)	O3-C39/O4-C58	1.294(3)/1.302(3)	O1-C1/O2-C20	1.294(5)/1.289(5)	O3-C39/O4-C58	1.293(5)/1.303(4)
C11-N1/C30-N2	1.299(3)/1.296(4)	C49-N3/C68-N4	1.288(3)/1.294(3)	C11-N1/C30-N2	1.286(5)/1.286(5)	C49-N3/C68-N4	1.285(5)/1.301(4)
O1-Co1-O2	109.7(1)	O3-Co2-O4	110.9(1)	O1-Co1-O2	109.5(1)	O3-Co2-O4	110.7(1)
N1-Co1-N2	123.9(1)	N3-Co2-N4	126.5(1)	N1-Co1-N2	124.1(1)	N3-Co2-N4	126.5(1)
O1-Co1-N1	94.1(1)	O3-Co2-N3	94.8(1)	O1-Co1-N1	94.0(1)	O3-Co2-N3	94.8(1)
O2-Co1-N2	94.2(1)	O4-Co2-N4	94.0(1)	O2-Co1-N2	93.8(1)	O4-Co2-N4	94.1(1)
O1-Co1-N2	118.7(1)	O3-Co2-N4	113.9(1)	O1-Co1-N2	117.5(1)	O3-Co2-N4	113.7(1)
O2-Co1-N1	117.3(1)	O4-Co2-N3	117.4(1)	O2-Co1-N1	118.9(1)	O4-Co2-N3	117.6(1)

[a] Two symmetry-independent molecules.

Table S3. Selected bond lengths (Å) and angles (°) in Λ-Co-*R*-L2 [(*R*)-**2**] and Δ-Co-*S*-L3 [(*S*)-**3**] (A and B).[a]

Λ-Co-*R*-L2		Δ-Co-*S*-L3 (A)		Δ-Co-*S*-L3 (B)	
Co1-O1/O3	1.924(3)/1.930(3)	Co1-O1	1.922(5)	Co2-O2	1.918(4)
Co1-N1/N2	1.982(3)/1.984(3)	Co1-N1	1.970(5)	Co2-N2	1.974(4)
O1-C7/O3-C23	1.306(5)/1.286(5)	O1-C1	1.303(9)	O2-C20	1.305(8)
C1-N1/C21-N2	1.295(5)/1.290(5)	C11-N1	1.297(7)	C30-N2	1.286(7)
O1-Co1-O3	116.8(2)	C16-Br1	1.895(6)	C35-Br2	1.894(6)
N1-Co1-N2	130.4(2)	O1-Co1-O1[ii]	117.8(3)	O2-Co2-O2[i]	118.4(3)
O1-Co1-N1	91.5(2)	N1-Co1-N1[ii]	125.7(3)	N2-Co2-N2[i]	126.9(3)
O3-Co1-N2	93.4(2)	O1-Co1-N1	92.6(2)	O2-Co2-N2	93.5(2)
O1-Co1-N2	111.9(2)	O1[ii]-Co1-N1	115.1(8)	O2[i]-Co2-N2	113.3(6)

[a] Two symmetry-independent molecules in Δ-Co-*S*-L3 [(*S*)-**3**], each with the Co atom on a special position. Symmetry transformations used to generate equivalent atoms: ii = $-x+2$, $-x+y+1$, $-z+2/3$ (A); y, x, $-z+1$ (B).

Table S4. Ligand chirality and substituents leading to chirality induction at-metal in *bis*[(*R*- or *S*)-N-1-(*p*-X-C_6H_4)ethyl-salicylaldiminato-κ^2*N*,*O*]-Λ- or Δ-M(II) in the solid state and in solution.

Complexes [#] [M(*R* or *S*-L)$_2$]	Ligand substituents (X)	Ligand chirality → induction at-metal at solid-state	Ligand chirality → induction at-metal in solution	Ref.
M = Co	H	*S* → Δ, *R* → Λ	*S* → Δ, *R* → Λ	5
	p-CH_3O	*S* → Δ, *R* → Λ	*S* → Δ, *R* → Λ	
	p-Cl or -Br	*S* → Δ, *R* → Λ	*S* → Δ, *R* → Λ	
M = Zn	H	*S* → Δ, *R* → Λ	*S* → Δ, *R* → Λ	6
	p-CH_3O	*S* → Δ, *R* → Λ	*S* → Δ, *R* → Λ	
	p-Cl or -Br	*S* → Δ, *R* → Λ	*S* → Δ, *R* → Λ	
M = Cu	H[a]	*S* → Λ, *R* → Δ	*S* → Λ, *R* → Δ	7
	p-CH_3O[a,b]	*S* → Δ, *R* → Λ	*S* → Λ, *R* → Δ	
	p-Cl or -Br[a]	*S* → Λ, *R* → Δ	*S* → Λ, *R* → Δ	
M = Ni[c]	*p*-CH_3O	*S* → Δ, *R* → Λ	*S* → Λ, *R* → Δ	8
	p-Cl	*S* → Λ, *R* → Δ	*S* → Λ, *R* → Δ	

[a] Opposite chirality induction at-metal with X = H, *p*-Cl or -Br in comparison to the homoleptic Co, Ni and Zn-complexes at solid-state and in solution, while similar induction with X = *p*-CH_3O at solid-state and opposite induction in solution.

[b] Solid *vs.* solution studies reveal chirality inversion at-metal.

[c] Dinuclear μ-aqua-*tetrakis*[(*R* or *S*)-*N*-1-(Ar)ethyl-salicylaldiminato]-di-Λ- or -Δ-nickel(II).

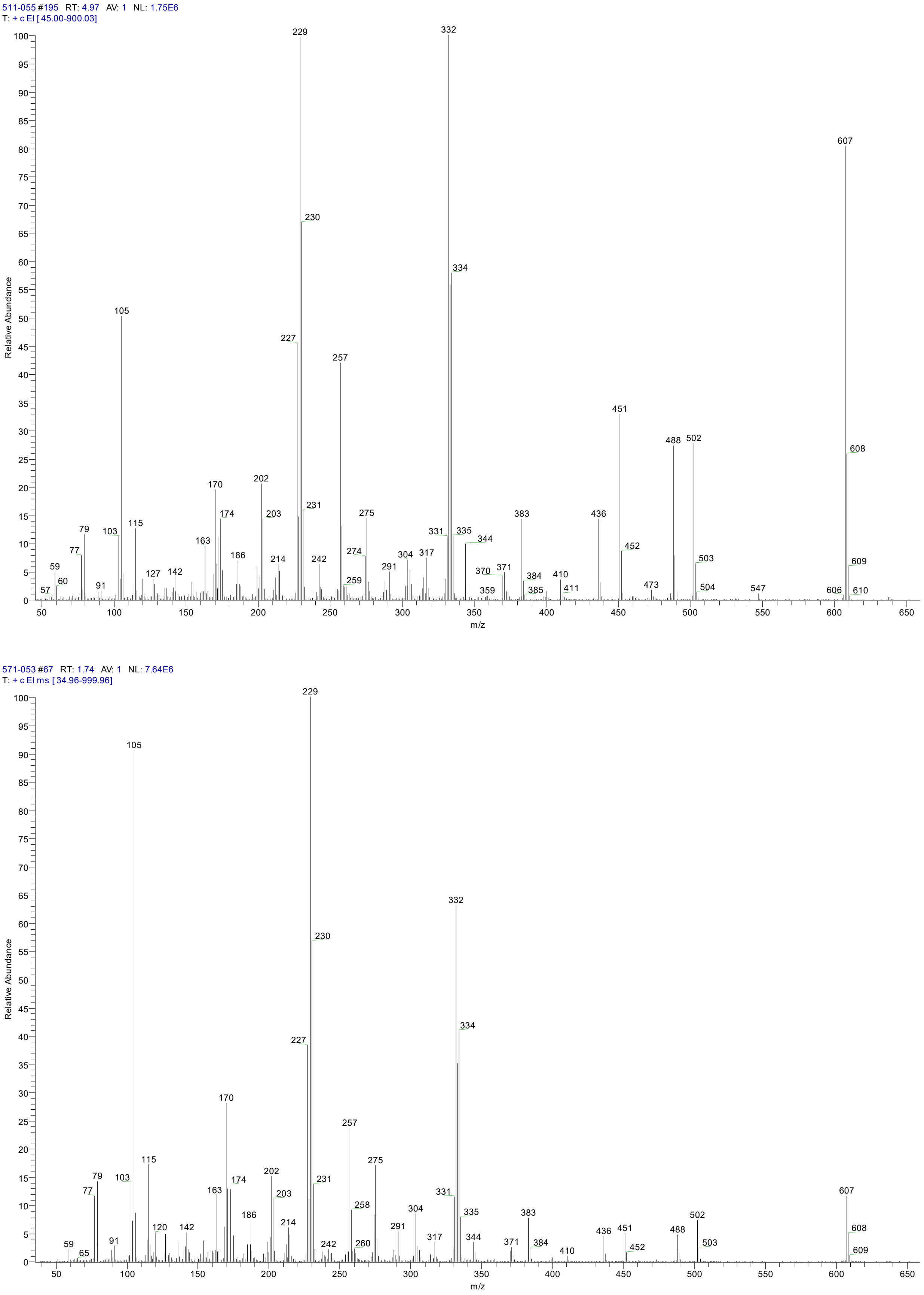

Figure S1. (a) EI-mass spectra for Co-*R*-L1 [(*R*)-**1**] (top) and Co-*S*-L1 [(*S*)-**1**] (bottom).

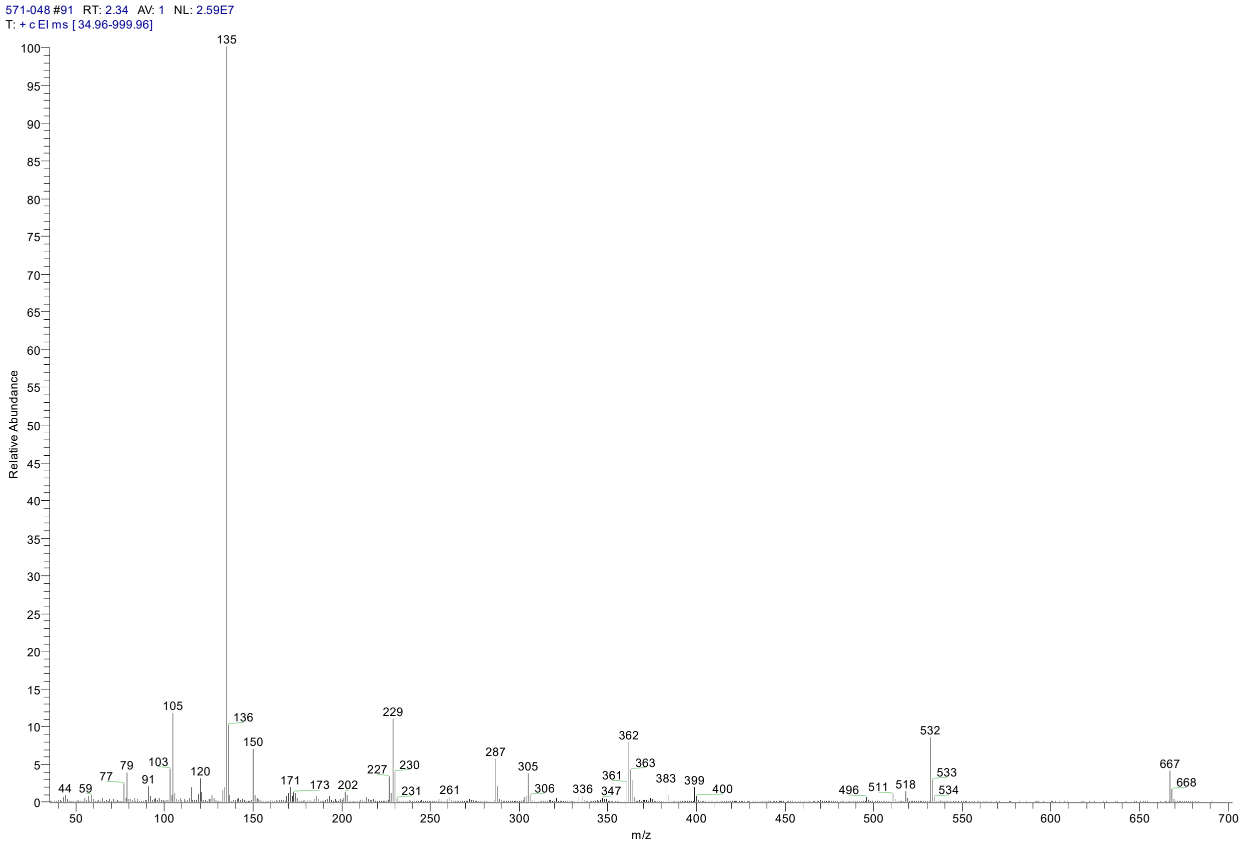

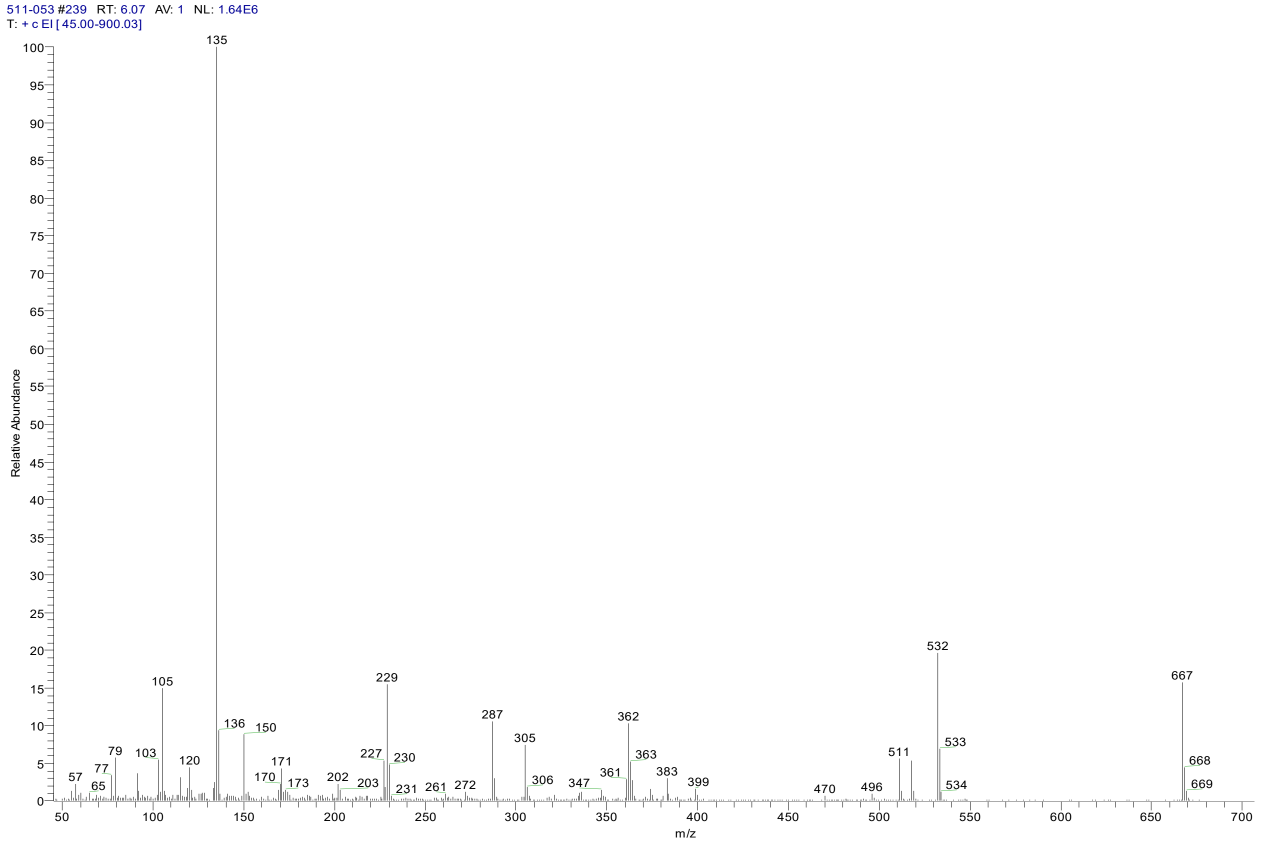

Figure S1. (b) EI-mass spectra for Co-*R*-L2 [(*R*)-**2**] (top) and Co-*S*-L2 [(*S*)-**2**] (bottom).

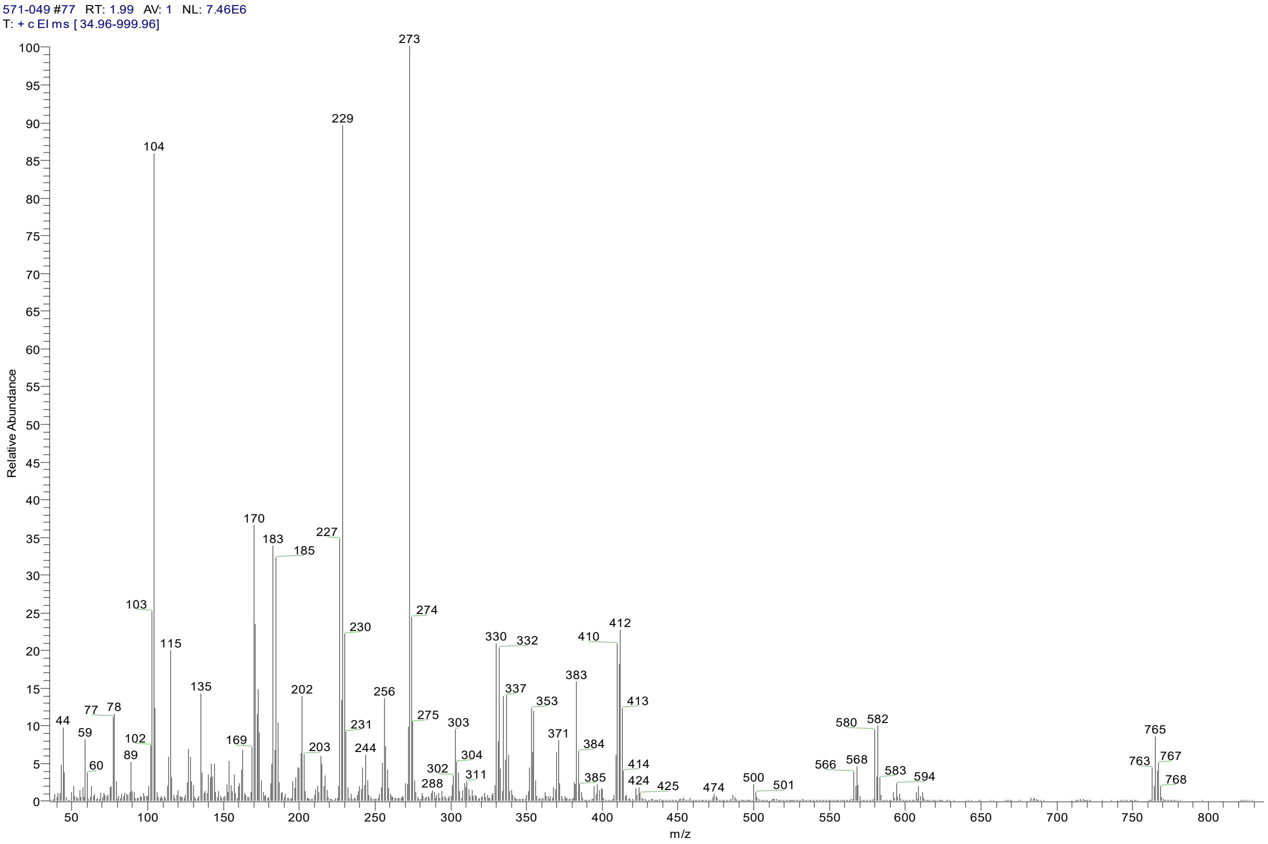

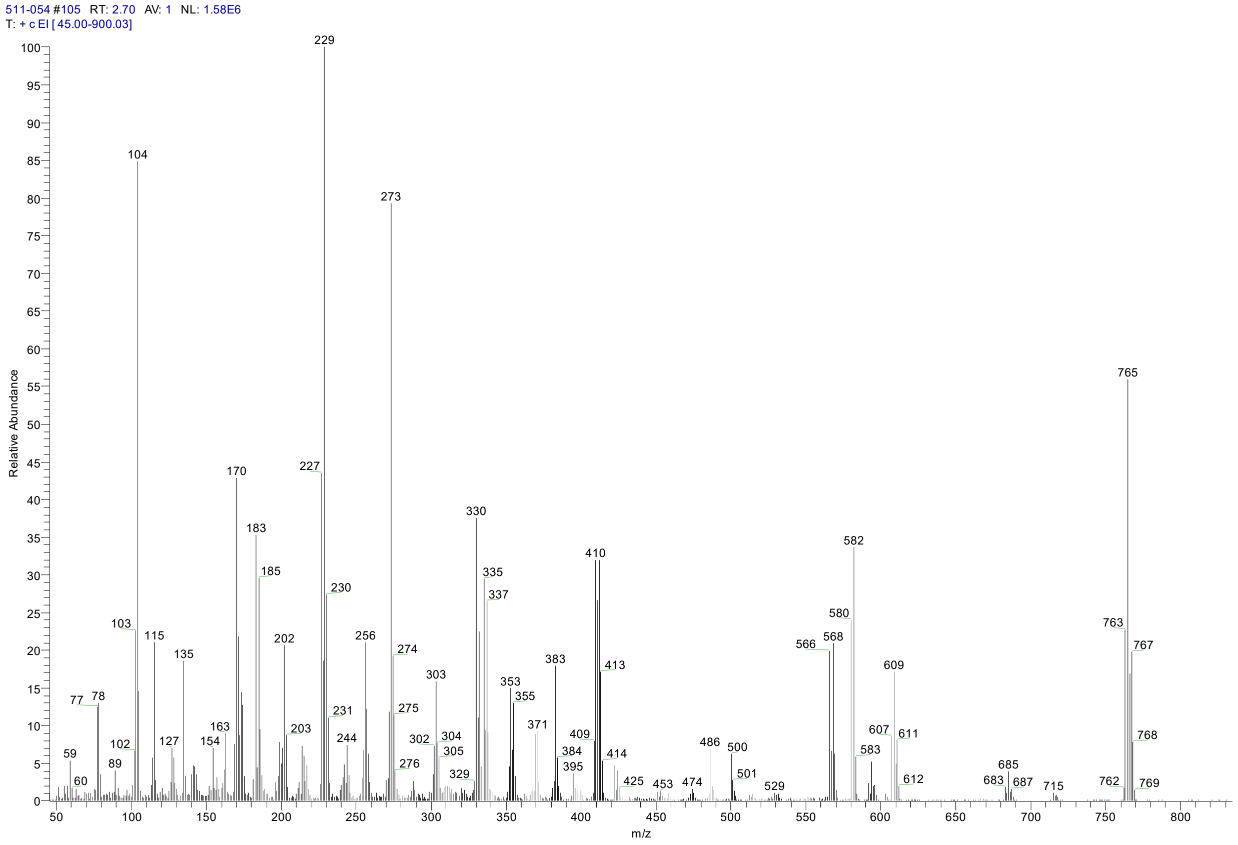

Figure S1. (c) EI-mass spectra for Co-*R*-L3 [(*R*)-**3**] (top) and Co-*S*-L2 [(*S*)-**3**] (bottom).

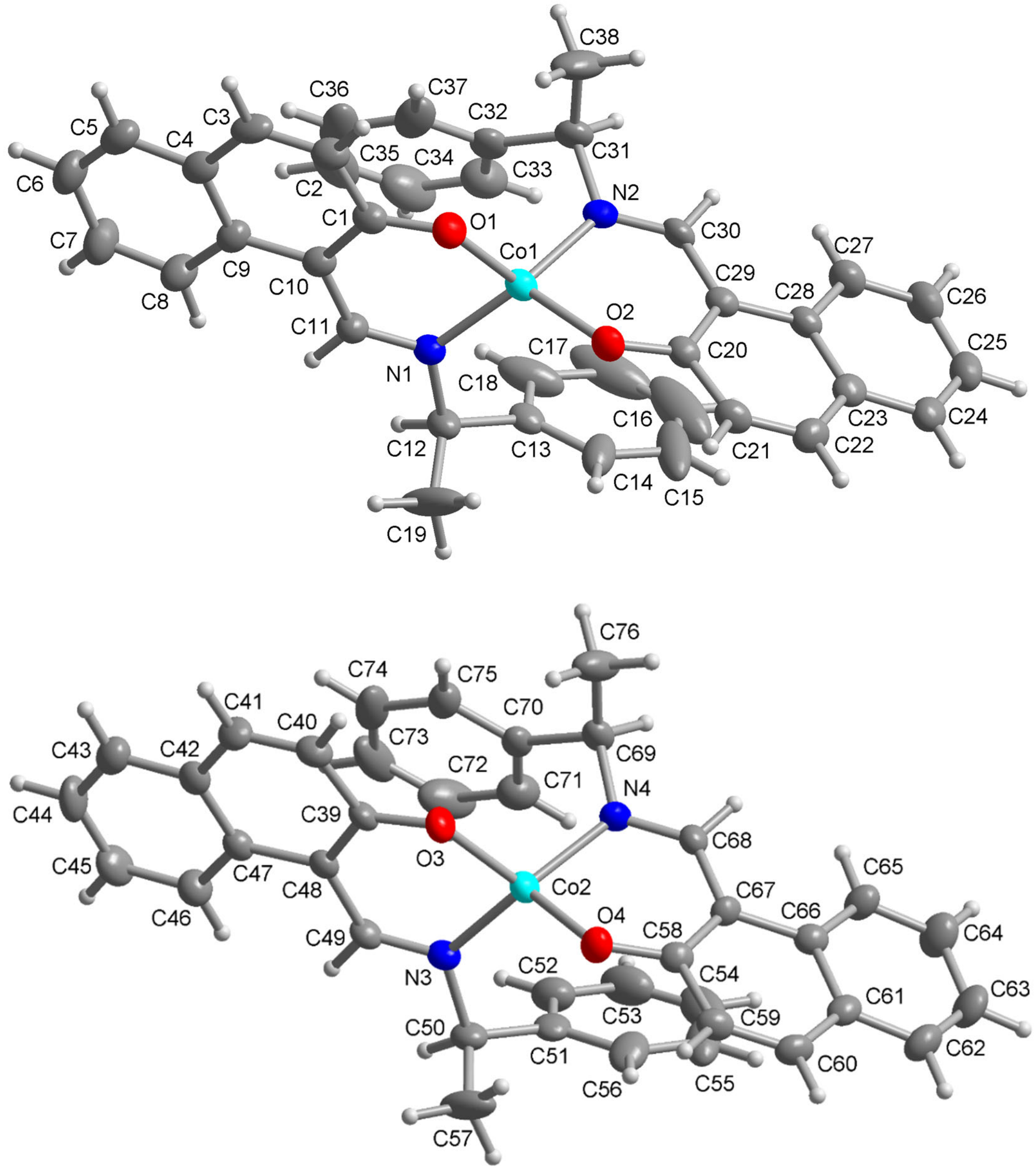

Figure S2. X-ray molecular structures of the symmetry-independent molecules in Δ-Co-*S*-L**1** [(*S*)-**1**] with full atom numbering scheme. Thermal ellipsoids at 50% (H atoms at arbitrary radii).

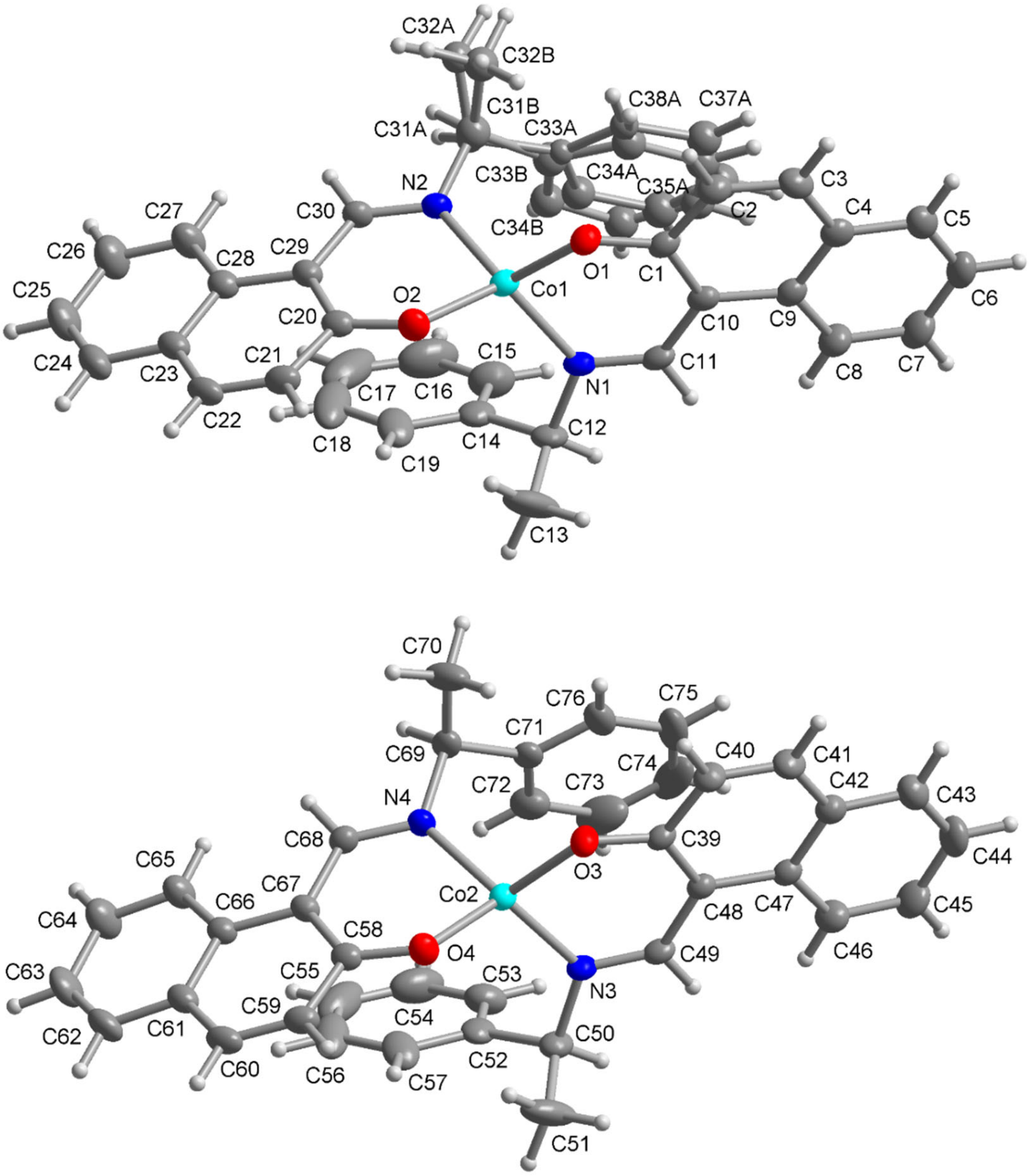

Figure S3. X-ray molecular structures of the symmetry-independent molecules in Λ-Co-*R*-L**1** [(*R*)-**1**] with full atom numbering scheme. Also shown is the disorder in one of the phenylethyl groups in molecule Co1 for which the atoms could only be refined isotropically. Thermal ellipsoids at 50% (H atoms at arbitrary radii).

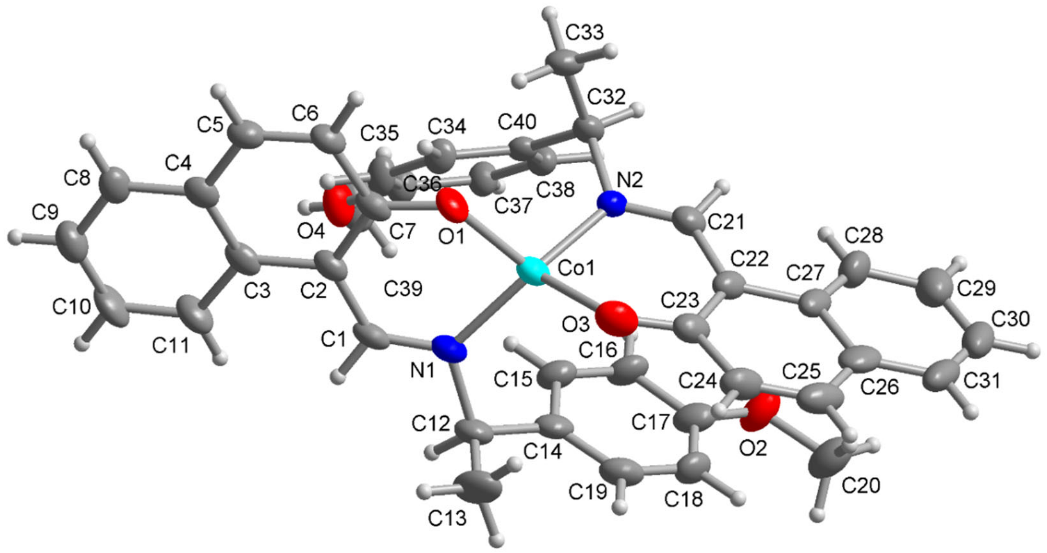

Figure S4. X-ray molecular structure in Λ-Co-*R*-L**2** [(*R*)-**2**] with full atom numbering scheme. Thermal ellipsoids at 50% (H atoms at arbitrary radii).

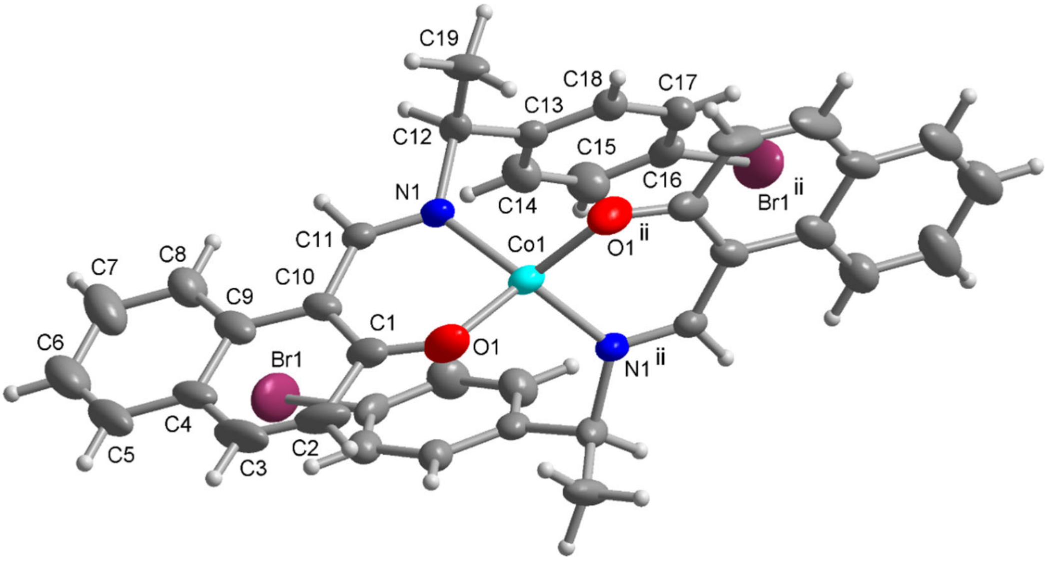

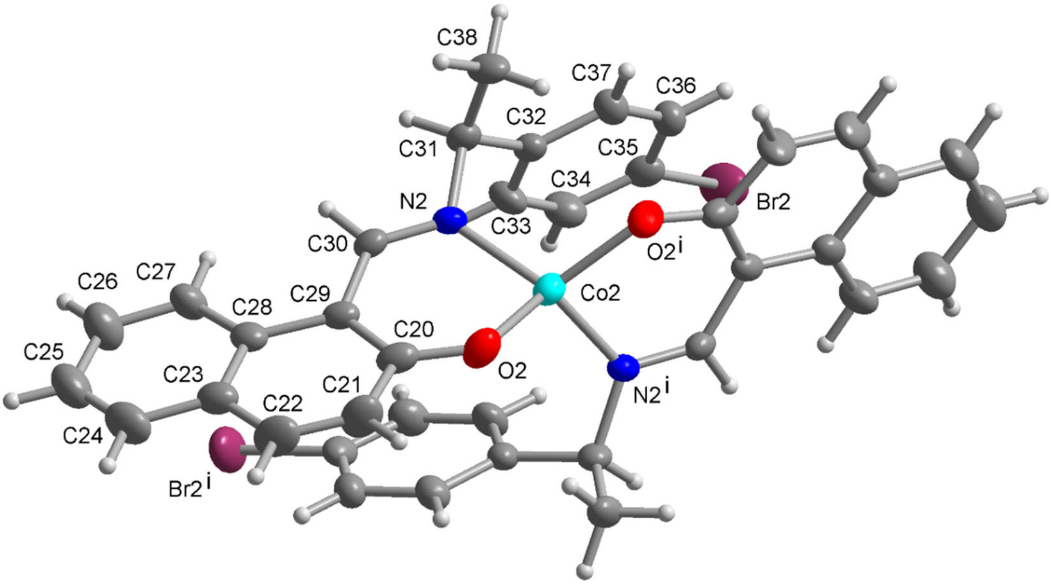

Figure S5. X-ray molecular structures of the symmetry-independent molecules in Δ-Co-*S*-L**3** [(*S*)-**1**] with full atom numbering scheme. Thermal ellipsoids at 50% (H atoms at arbitrary radii). Symmetry labels: i = y, x, 1–z; ii = 2–x, 1–x+y, 2/3–z.

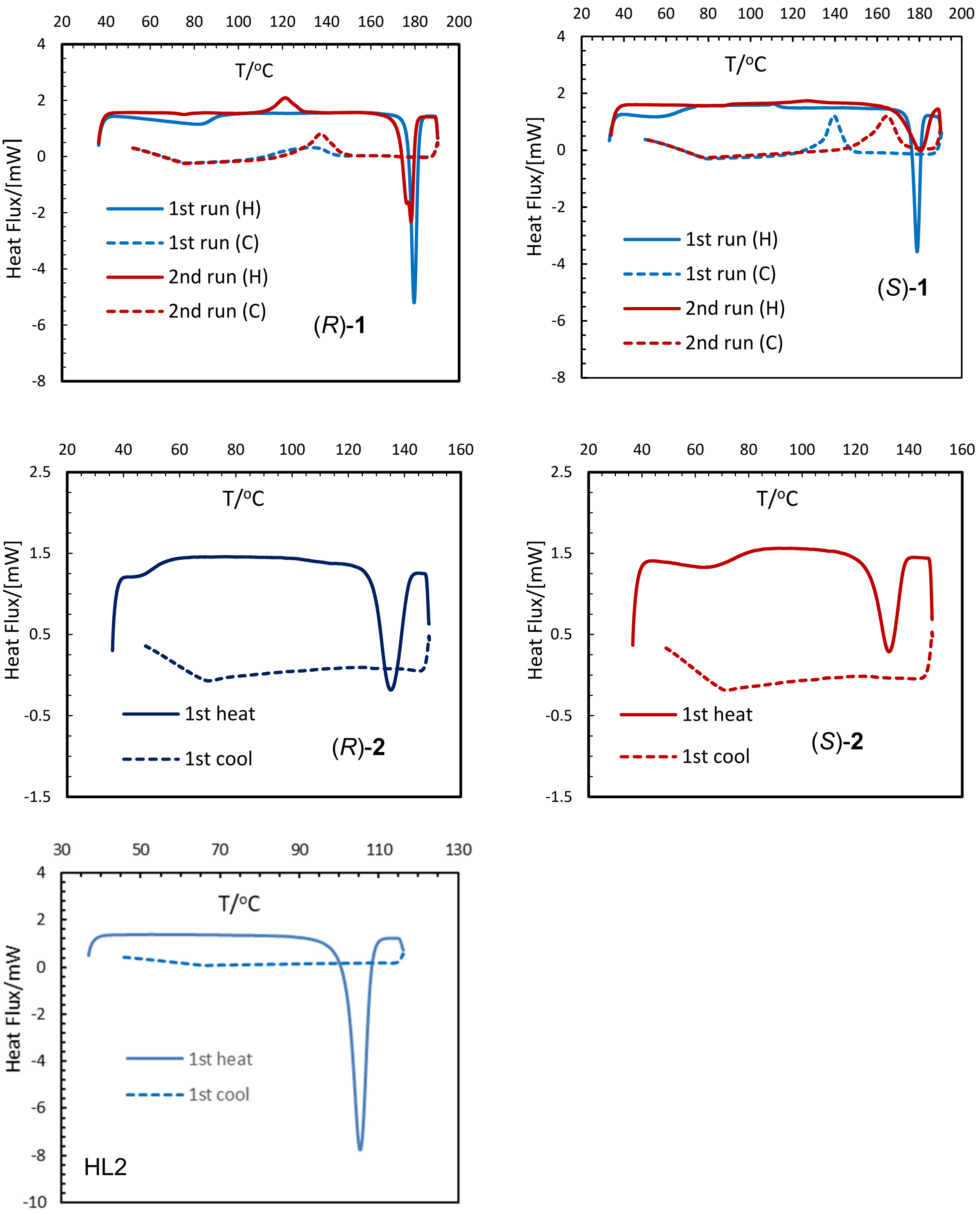

Figure S6. Differential Scanning Calorimetry (DSC) curves for complexes (*R*)-**1**, (*S*)-**1**, (*R*)-**2**, and (*S*)-**2**, and for ligand HL2 (H/C = heating/cooling).

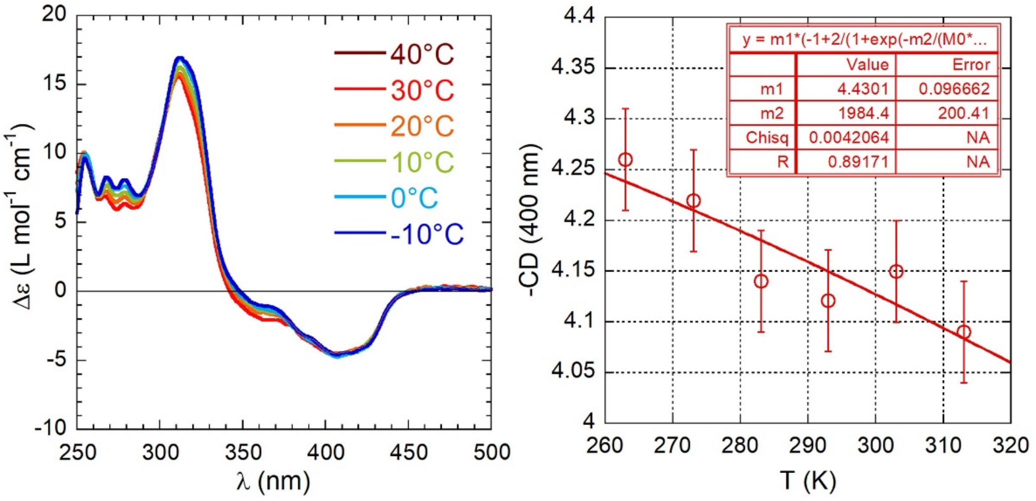

Figure S7. Left: variable-temperature ECD spectra of (*S*)-**1** in $CHCl_3$. Right: fitting of ECD value at 400 nm according to the equation describing a single apparent equilibrium between two species:

$$Y = m_1\left(\frac{2}{1+\exp\left(-m_2/RT\right)}-1\right)$$

where parameter m_1 is the molar ECD of the major species at 400 nm (Δ isomer), considered to be equal in intensity and opposite in sign of the ECD of the minor species (Λ isomer), and parameter m_2 is the apparent ΔG° of the process in cal/mol (assumed to be temperature-independent over the observed range).

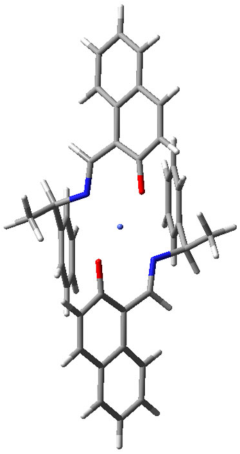	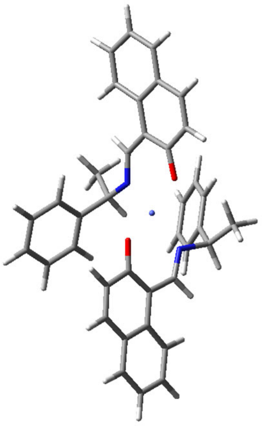	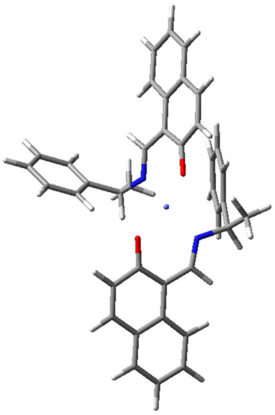	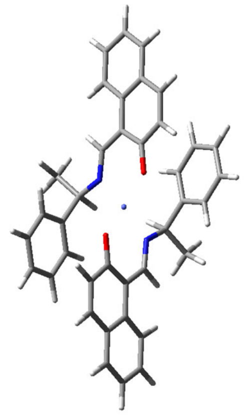
Λ-(*R*)<br>lowest energy	Λ-(*R*)<br>+1.52 kcal/mol	Λ-(*R*)<br>+4.30 kcal/mol	Λ-(*R*)<br>+7.17 kcal/mol
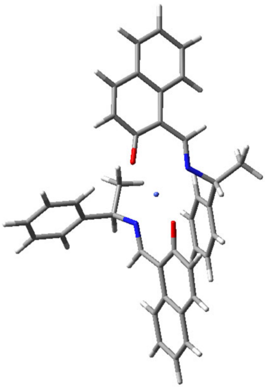	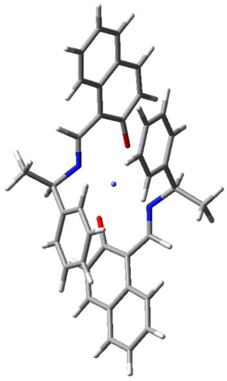	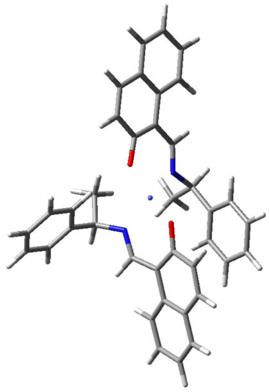	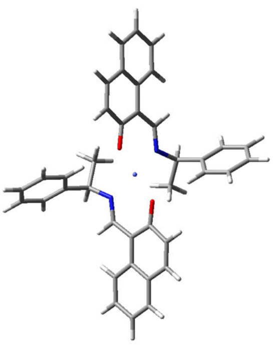
Δ-(*R*)<br>+3.71 kcal/mol	Δ-(*R*)<br>+3.91 kcal/mol	Δ-(*R*)<br>+4.07 kcal/mol	Δ-(*R*)<br>+4.40 kcal/mol
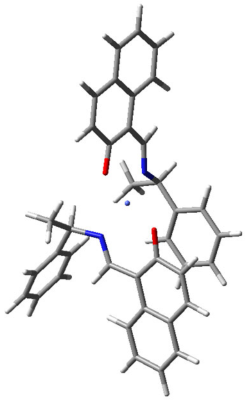	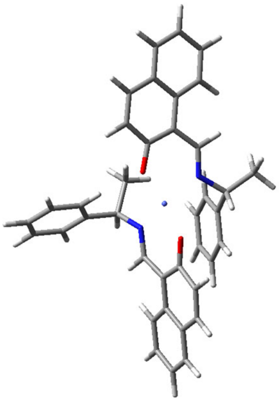	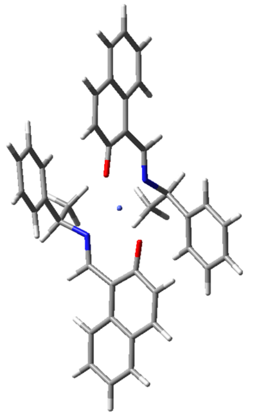	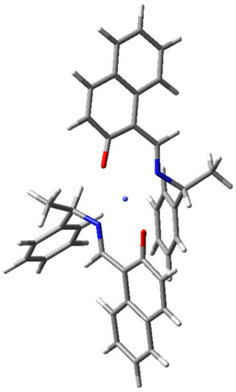
Δ-(R)<br>+4.67 kcal/mol	Δ-(R)<br>+5.03 kcal/mol	Δ-(R)<br>+5.40 kcal/mol	Δ-(R)<br>+5.66 kcal/mol

Figure S8. Geometries and relative energies calculated for (*R*)-**1** at B3LYP-D3/def2-TZVP/PCM //B3LYP/def2-SVP level with PCM for chloroform. First Λ-(R) conformers are listed, then Δ-(R) conformers, with relative energies < 7.25 kcal/mol from the absolute minimum.

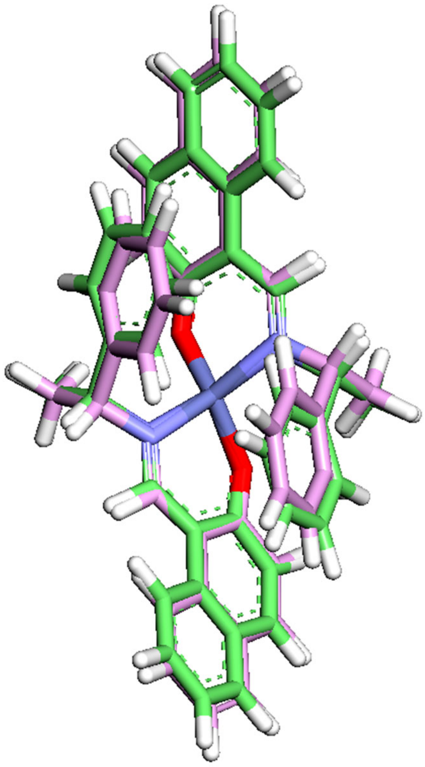

Figure S9. Molecular overlay between the X-ray structure of (*R*)-**1** (green) and the lowest-energy DFT-optimized structure (pink). Calculations run at B3LYP-D3/def2-TZVP level with PCM for chloroform.

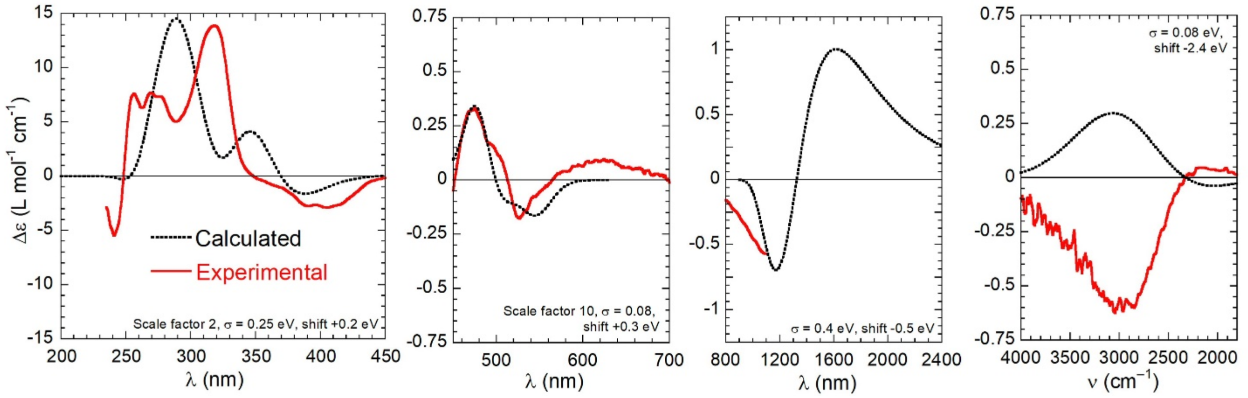

Figure S10. Comparison between experimental (red lines) and calculated (black dotted lines) CD superspectra of (*R*)-**1**. Calculations run at the B3LYP/def2-TZVP//B3LYP-D3/def2-TZVP level with PCM for chloroform, as Boltzmann averages over two Λ conformers. The parameters used to generate the calculated spectra in each region are given as insets.

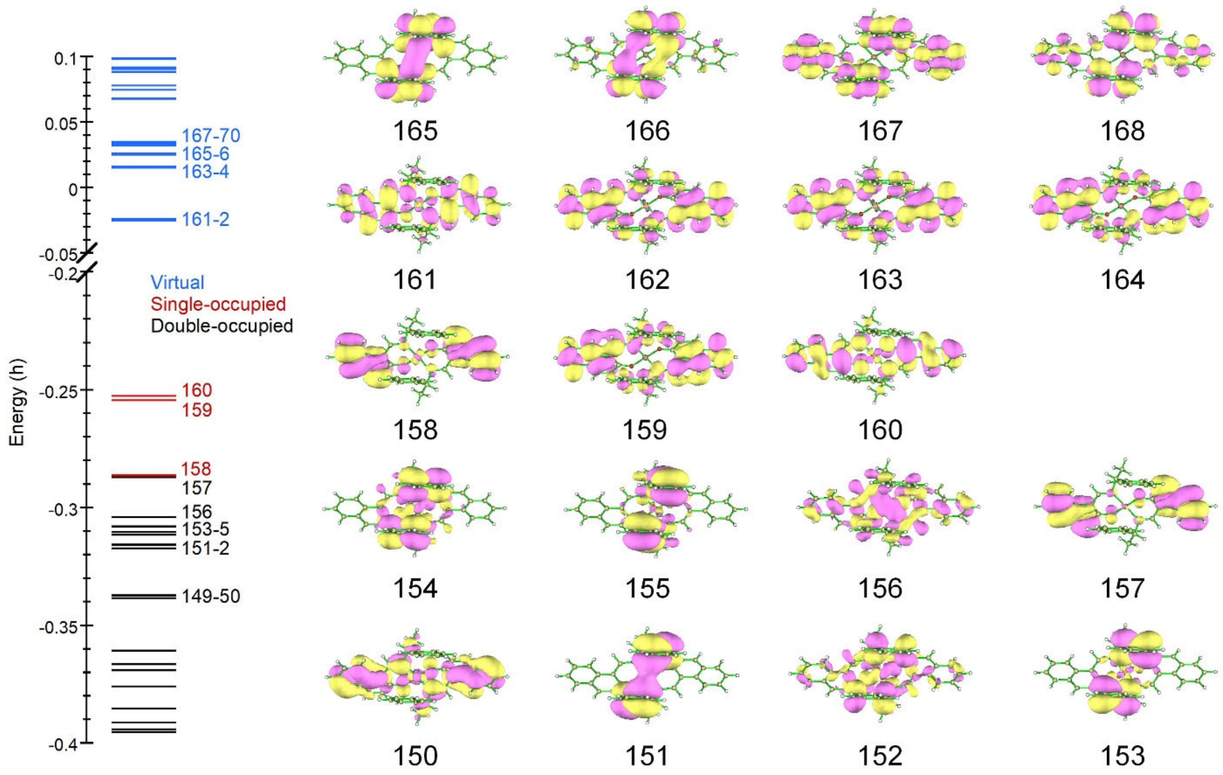

Figure S11. Frontier Kohn-Sham molecular orbitals (MO) and energies calculated at the CAM-B3LYP/ def2-TZVP//B3LYP-D3/ def2-TZVP level for C_2-symmetric structure of (*R*)-**1**. Plotted with MultiWfn v3.4;[9] isovalue 0.02.

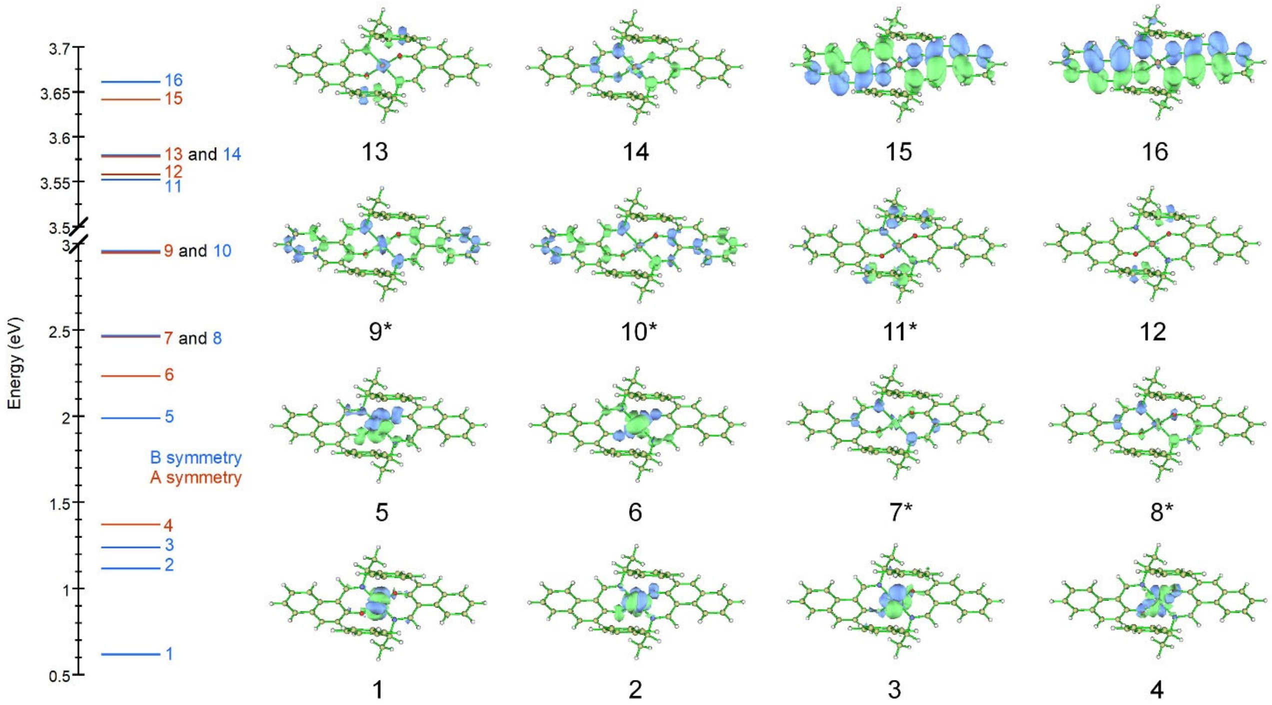

Figure S12. Transition energies, symmetries and density plots calculated at the CAM-B3LYP/def2-TZVP//B3LYP-D3/ def2-TZVP level for C_2-symmetric structure of (*R*)-**1**. Plotted with MultiWfn v3.4;[9] isovalue 0.001 or 0.0005 (*).

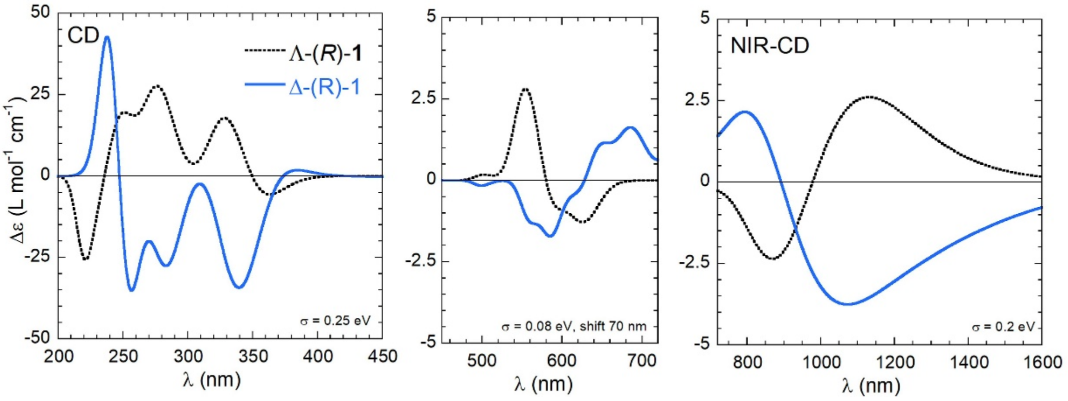

Figure S13. Comparison of ECD spectra calculated at the CAM-B3LYP/def2-TZVP level with PCM for chloroform, as Boltzmann average on the two lowest-energy Λ-(*R*)-**1** conformers, and on the four lowest energy Δ-(*R*)-**1** conformers (see Figure S8). Geometries optimized at B3LYP/def2-SVP level and populations estimated at B3LYP-D3/def2-TZVP level with PCM for chloroform.

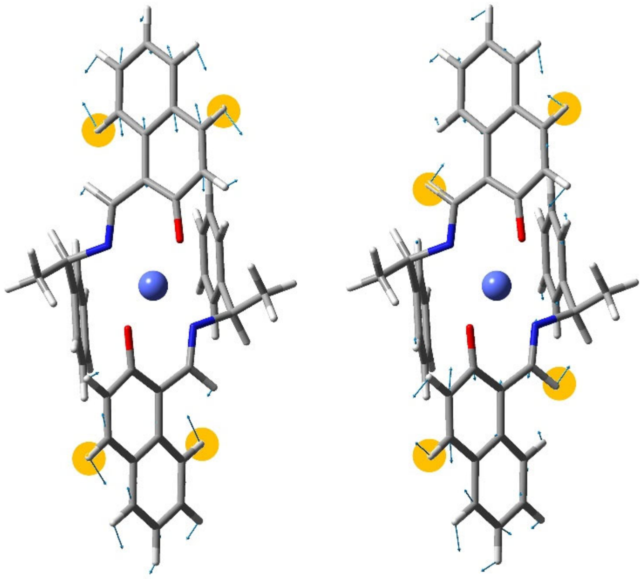

Figure S14. Atom displacements (light blue arrows) for B-symmetry normal modes 182 and 186; the H atoms most contributing to the normal modes are highlighted.

References for the Supporting Information

1. Flack, H. On enantiomorph-polarity estimation. *Acta Cryst. A* **1983**, *39*, 876-881.
2. Flack, H. D.; Bernardinelli, G. Absolute structure and absolute configuration. *Acta Cryst. A* **1999**, *55*, 908-915.
3. Flack, H. D.; Bernardinelli, G. The use of X-ray crystallography to determine absolute configuration. *Chirality* **2008**, *20*, 681-690.
4. Flack, H. D.; Sadki, M.; Thompson, A. L.; Watkin, D. J. Practical applications of averages and differences of Friedel opposites. *Acta Cryst. A* **2011**, *67*, 21-34.
5. Pescitelli, G.; Lüdeke, S.; Chamayou, A. C.; Marolt, M.; Justus, V.; Gorecki, M.; Arrico, L.; Di Bari, L.; Islam, M. A.; Gruber, I.; Enamullah, M.; Janiak, C. Broad-Range Spectral Analysis for Chiral Metal Coordination Compounds: (Chiro)optical Superspectrum of Cobalt(II) Complexes. *Inorg. Chem.* **2018**, *57*, 13397-13408.
6. Enamullah, M.; Vasylyeva, V.; Janiak, C. Chirality and diastereoselection of Δ/Λ-configured tetrahedral zinc(II) complexes with enantiopure or racemic Schiff base ligands. *Inorg. Chim. Acta* **2013**, *408*, 109-119.
7. Chamayou, A. C.; Makhloufi, G.; Nafie, L. A.; Janiak, C.; Lüdeke, S. Solvation-Induced Helicity Inversion of Pseudotetrahedral Chiral Copper(II) Complexes. *Inorg. Chem.* **2015**, *54*, 2193-2203.
8. Enamullah, M.; Quddus, M. A.; Hasan, M. R.; Pescitelli, G.; Berardozzi, R.; Reiss, G. J.; Janiak, C. Syntheses, Spectroscopy, and Structural Analyses of Dinuclear Chiral-at-Metal -Aqua-tetrakis (R or S)-N-1-(Ar)ethylsalicylaldiminato di-Λ- or -Δ-nickel(II) Complexes. *Eur. J. Inorg. Chem.* **2015**, 2758-2768.
9. Lu, T.; Chen, F. Multiwfn: A multifunctional wavefunction analyzer. *J. Comput. Chem.* **2012**, *33*, 580-592.
